# Biocontrol of Cheese Spoilage Moulds Using Native Yeasts

**DOI:** 10.3390/foods14142446

**Published:** 2025-07-11

**Authors:** Catalina M. Cabañas, Alejandro Hernández León, Santiago Ruiz-Moyano, Almudena V. Merchán, José Manuel Martínez Torres, Alberto Martín

**Affiliations:** 1 Departamento de Producción Animal y Ciencia de los Alimentos, Nutrición y Bromatología, Escuela de Ingenierías Agrarias, Universidad de Extremadura, Avd. Adolfo Suárez s/n, 06007 Badajoz, Spain; cmcabanas@unex.es (C.M.C.); ahernandez@unex.es (A.H.L.); avmerchan@unex.es (A.V.M.);; 2 Instituto Universitario de Investigación en Recursos Agrarios (INURA), Universidad de Extremadura, Avd. de la Investigación s/n, 06006 Badajoz, Spain; 3 Departamento de Producción Animal y Ciencia de los Alimentos, Producción Animal, Escuela de Ingenierías Agrarias, Universidad de Extremadura, Avda. Adolfo Suárez s/n, 06007 Badajoz, Spain; jmtorres@unex.es

**Keywords:** yeast, biocontrol, cheese, *Pichia jadinii*, *Geotrichum candidum*, spoilage moulds

## Abstract

Biocontrol is one of the most promising alternatives to chemical preservatives for food preservation. This study investigated the biocontrol potential of yeasts isolated from raw milk cheese against spoilage moulds. Eighty-four native yeast strains were screened for antagonistic activity against *Penicillium commune*, *Fusarium verticillioides*, and *Mucor plumbeus/racemosus* via confrontation using a milk-based culture medium. Fifteen strains from the species *Pichia jadinii*, *Kluyveromyces lactis*, *Kluyveromyces marxianus*, and *Geotrichum candidum* exhibited significant antagonistic activity (inhibition zone > 2 mm) against *M. plumbeus/racemosus* and *F. verticillioides*. The modelling of the impact of ripening conditions revealed that temperature was the primary factor influencing yeast antagonism. In addition, notable variability at both species and strain levels was found. The antagonist activity was associated with different mechanisms depending on the species and strains. *K. lactis* stood out for its proteolytic activity and competition for iron and manganese. Additionally, two strains of this species (KL890 and KL904) were found to produce volatile organic compounds with antifungal properties (phenylethyl alcohol and 1-butanol-3-methyl propionate). *G. candidum* GC663 exhibited strong competition for space, as well as the ability to parasitise hyphae linked to its pectinase and β-glucanase activity. The latter enzymatic activity was detected in all *P. jadinii* strains, with *P. jadinii* PJ433 standing out due to its proteolytic activity. In a cheese matrix, the efficacy of eight yeast strains against three target moulds was assessed, highlighting the potential of *G. candidum* GC663 and *P. jadinii* PJ433 as biocontrol agents, exhibiting high and moderate efficacy, respectively, in controlling the growth of *F. verticillioides* and *M. plumbeus/racemosus*. Nonetheless, further research is necessary to elucidate their full spectrum of antifungal mechanisms and to validate their performance under industrial-scale conditions, including their impact on cheese quality.

## 1. Introduction

The microbiota of traditional cheeses is mainly composed of lactic acid bacteria (LAB) and, to a lesser extent, Gram-positive catalase-positive bacteria, members of the Enterobacteriaceae family, *Pseudomonas* spp., and fungi [1]. Within this diverse microbial community, moulds are ubiquitous and can contaminate cheese surfaces at any stage of production, acting as common spoilage organisms [2]. Mould’s population in traditional cheeses is highly diverse and varies significantly between cheese types. *Penicillium* and *Mucor* are the most prevalent genera, while *Cladosporium*, *Aspergillus*, and *Fusarium* are also commonly encountered, albeit to a lesser degree [3]. The development of spoilage moulds can lead to various types of spoilage, including visible growth, changes in colour and texture, and the formation of off-flavours such as excessive mustiness or bitterness [4]. These aspects contribute to a shortened shelf life, diminished consumer satisfaction, and substantial economic losses. Furthermore, mould proliferation in cheese may result in potential mycotoxin contamination, including ochratoxin A, patulin, cyclopiazonic acid (CPA), mycophenolic acid, and roquefortine C [5], posing significant risks to human health.

Currently, preventive and control approaches are often used together to avoid or limit mould growth during the cheese-making process. Preventive measures, such as good manufacturing practices (GMPs), Hazard Analysis Critical Control Point (HACCP) systems, and air and equipment decontamination, aim to prevent microbial contamination, while control strategies focus on retarding or inhibiting mould growth. Among the latter, the most effective and common is the use of chemical preservatives on the cheese surface. These include natamycin and organic acids such as propionic and sorbic, as well as their salts [3,6]. However, legislation is becoming increasingly restrictive regarding their use and authorised dose due to the emergence of resistant mould species, the negative impact on sensory quality, and environmental drawbacks. Notably, resistance to sorbates has been reported among species of the *Penicillium* genus [7,8]. Moreover, some moulds can metabolise sorbates via decarboxylation, producing plastic-like or ‘kerosene’ off-flavour in cheeses [9]. Similarly, effective propionate concentrations can negatively impact the sensory properties of the product [10]. For these reasons, natamycin is frequently preferred over other preservatives, being approved in most countries for the antifungal surface treatment of cheese. Its key advantages include being odourless, colourless, and exhibiting a low incidence of fungal resistance [11]. However, fungi could develop resistance to preservatives over time. For example, high-level resistance conferred by a natamycin-degrading enzyme has recently been documented in *Penicillium discolor* [12]. In addition, today there is a growing demand for less processed and preservative-free foods with high quality and safety standards [13]. In this scenario, the food industry has intensified the search for natural alternatives to chemical preservatives. In the dairy industry, biopreservation, which involves the use of microorganisms and/or their antimicrobial metabolites, has attracted particular attention for controlling spoilage fungi [6].

In cheese environments, biopreservation using LAB has been the primary strategy investigated. This microbial group plays a key role in cheese ripening, has GRAS (Generally Recognised as Safe, in the US) and QPS (Qualified Presumption of Safety, in the EU) status, and can produce several antifungal metabolites, including organic acids, fatty acids, hydrogen peroxide, bacteriocins, reuterin, and some volatile compounds [14,15,16]. This has enabled the commercial availability of some LAB strains with verified antifungal activity for cheese applications (e.g., Delvo^®^Guard (DSM), Lyofast LPR A (SACCO), Lyopro^®^ Tect (Codex-ing), HOLDBAC^®^YM-XPM (Danisco), and FreshQ^®^ (CHR Hansen) [17]. However, other microorganisms present in the cheese matrix remain underexplored for this purpose. Among these, yeasts hold significant potential as bioprotective cultures. Their simple cultivation, established biosafety (except certain *Candida* and *Cryptococcus* species), adaptation to the cheese surface environment, known contribution to flavour, and potential antifungal properties (e.g., killer toxin, space and nutrient competition, antifungal volatile organic compounds, lytic enzymes, and mycoparasitism) against moulds make them particularly attractive candidates [18,19]. Although their main application as biocontrol agents has been against plant pathogenic moulds [20], certain cheese-associated species from dairy and other origins have shown promise in this area. *Debaryomyces hansenii* has demonstrated strong inhibitory activity against the dairy and dry-cured meat spoilage moulds *Cladosporium* spp., *Aspergillus* spp., *Byssochlamys fulva*, *Penicillium candidum*, *Penicillium roqueforti*, and *Penicillium nordicum* [21,22,23]. Similarly, *Pichia kudriavzevii*, *Kluyveromyces lactis*, and *Kluyveromyces marxianus* strains from various sources can control the growth of toxigenic moulds, including *Aspergillus flavus*, *Aspergillus parasiticus*, and *Penicillium expansum* [24,25,26]. In mould-ripened cheese, interactions between *D. hansenii* and *Yarrowia lipolytica* have been reported to limit the growth of *Penicillium camemberti* and *P. roqueforti*, respectively [27,28]. Therefore, selecting yeast strains with a biocontrol capacity against cheese spoilage moulds is of significant interest to the dairy industry. Accordingly, this study aimed to determine and characterise the antagonist activity of yeast strains isolated from raw milk soft cheeses against three spoilage moulds (*Penicillium commune*, *Mucor plumbeus/racemosus,* and *Fusarium verticillioides*) and, subsequently, evaluate the efficacy of the most promising strains on cheese wedges.

## 2. Materials and Methods

### 2.1. Yeast and Mould Strains

A total of 84 yeast and 3 spoilage mould strains were used in this survey (Supplemental Table S1). The yeasts used were isolated from soft raw ewe’s milk and genetically identified at the strain level in our previous study [29]. For identification, inter-single sequence repeat-anchored PCR amplification using primer (GTG)^5^ was used, followed by ITS genetic marker sequencing. To obtain the spoilage moulds, cheese rind samples with the occurrence of mould were taken at the end of the ripening period from 6 different dairies (3 dairies belonging to PDO Queso de la Serena and the other 3 to PDO Torta del Casar). Samples were diluted 10-fold with 1% peptone water (Condalab, Madrid, Spain) and homogenised for 120 s in a stomacher (Lab Blender 400, Seward Medical, London, UK). The homogenates were serially diluted and aliquots from each dilution were inoculated on acidified potato dextrose agar plates (PDA; Condalab) with a 1% (*v*/*v*) sterilised solution of tartaric acid at 10% (*w*/*v*). PDA plates were incubated at 25 °C for 5 days. The isolation of moulds was performed based on the colony morphology. Two colonies of each morphology and sample were selected from the highest dilutions. Each isolate was subcultured on PDA until it became a pure colony. After this, the isolates were stored in 25% sterile glycerol (*v*/*v*) at −80 °C.

To identify the moulds at the species level, the genomic DNA from mould isolates was extracted using the NucleoSpin^®^ Microbial DNA kit (Macherey–Nagel, Düren, Germany) according to the manufacturer’s instructions. Genetic markers, ITS, β-tubulin and 26S large ribosomal subunit, were amplified using the primer pairs ITS1/ITS4 [30], Bt2a/Bt2b [31], and NL1/NL4 [32], respectively. PCR reactions were performed according to the reactive concentrations and conditions described according to Cabañas et al. [33]. The PCR products obtained were purified using the GeneJET PCR purification kit (Thermo Fisher Scientific, Waltham, MA, USA) and then sequenced at the Service of Bioscience Applied Techniques (STAB) of the University of Extremadura (Badajoz, Spain). The sequences were edited using Bioedit version 7.2 and checked byvia nucleotide–nucleotide BLAST comparison in the NCBI database. The identities of the isolates were determined based on the highest score. Moreover, for the specific identification of isolates belonging to the *P. commune* and *Penicillium biforme* species, CPA production was determined after growth on Yeast Extract Sucrose (YES, Condalab; 7 days at 25 °C) agar via micellar electrokinetic capillary chromatography following the methodology described according to Casquete [34].

### 2.2. Microorganism Culture and Inoculum Preparation

Before the assays, all yeast strains were subcultured twice in yeast extract peptone dextrose agar (YPD; Condalab) at 25 °C for 48 h. Subsequently, a loop of cells was collected and resuspended in 1 mL of sterile distilled water. The yeast concentration was determined using a Neubauer chamber and adjusted to 10^7^ cells/mL.

Regarding moulds, the target strains were routinely grown on PDA at 25 °C for around 10 days until sporulation. The spores were harvested by adding 10 mL of sterile water containing 0.05% (*v*/*v*) Tween 80 (Scharlab, Barcelona, Spain) to each mould plate. The surface of each colony was gently swept, and the suspension obtained was filtered through two layers of cheesecloth. The concentration of the spore suspension was determined using a Neubauer chamber and adjusted to 10^5^ spores/mL with sterile distilled water.

### 2.3. Screening of Antagonistic Activity

*M. plumbeus/racemosus* 2367, *P. commune* 1031, and *F. verticillioides* 1191 were used as target cheese spoilage moulds to screen the antagonistic activity of selected yeasts. The antagonistic capability of yeast strains against the three target moulds was determined via a direct confrontation assay between the yeasts and the spoilage moulds. The PDA and skim milk (SM) agar adjusted at different conditions of pH, NaCl, and a_w_ were used for the assay. The SM medium was prepared by dissolving skim milk powder (50 g/L; PanReac AppliChem, Barcelona, Spain) and bacteriological agar (15 g/L; Condalab) in distilled water, sterilising it via autoclaving for 5 min at 115 °C, and then pouring it onto Petri dishes.

To evaluate the influence of pH, the media were adjusted to pH 4.5, 5, and 5.5 after autoclaving with a sterilised solution of lactic acid at 10% (*w*/*v*). Next, to analyse the influence of NaCl on the antagonist activity, media adjusted to pH 5.5 were used and supplemented with different concentrations of NaCl (0, 1.5, and 3%). Finally, the influence of a_w_ was evaluated in media at pH 5.5, 3% NaCl, and variable a_w_ (0.97, 0.95, and 0.92). The a_w_ of the media was modified by the addition of appropriate amounts of glycerol (Scharlab). To evaluate the antagonistic capacity of yeast, 100 μL from a spore solution of each target mould was added to the agar surface and spread out. After drying, 5 μL of each yeast suspension was spotted onto agar plates at equidistant positions. As controls, the test was carried out in the same manner using 5 μL of sterile distilled water instead of yeast suspension. The plates were incubated for 5 days at 25 °C. Modifications in the growth of target moulds on the yeast plates were compared to the control plates. The confrontations were performed in triplicate. Yeast strains exhibiting the greatest effect on mycelial growth were selected to model the influence of the cheese ripening conditions on their antifungal activity and to characterise the mechanism of action.

### 2.4. Modelling the Impact of Cheese Ripening Conditions on the Antifungal Activity

A Box–Behnken design (BBD) with three factors was used to model the combined effects of temperature, a_w_, and pH on the antagonist activity of the yeasts selected in Section 2.3 as a function of their ability to produce halos. The variable factor conditions are shown in Supplemental Table S2 and were established according to the physicochemical and ripening conditions commonly found in traditional cheeses [35,36]. Skimmed milk agar medium was used for the test. The pH and a_w_ of the SM agar containing NaCl at 2.5% (*p*/*v*) were set up at the values shown in Supplemental Table S2, as described in Section 2.3. Afterwards, 100 μL of a solution of spore of each target mould was added to the surface and spread. After drying, 5 μL of each yeast suspension was inoculated onto the SM agar plates in equidistant positions. After inoculation, the SM agar plates were incubated at different temperatures ranging from 8 °C to 20 °C (Supplemental Table S2) for 7 days. Once the moulds had grown, the radio of the halos that had formed was recorded.

### 2.5. Characterisation of the Mechanism of Antagonist Activity

#### 2.5.1. In Vitro Biofilm Formation

The capacity to form biofilm in vitro was determined, as detailed according to Cordero-Bueso et al. [37]. A positive control strain, *K. marxianus* 2459, from the collection of the CAMIALI group from the University of Extremadura was also used as a positive control strain. The determination was performed in triplicate.

#### 2.5.2. Production of Antifungal Extracellular Substances: Antibiosis and Lytic Enzyme

##### Antibiosis

Yeasts selected for their ability to produce an inhibition halo were inoculated at a final concentration of 10^5^ cells/mL in SM broth adjusted to pH 5.5 in the presence and absence of the target moulds (*M. plumbeus/racemosus* 2367 and *F. verticillioides* 1191) in triplicate. The mould was inoculated at a final concentration of 10^3^ spore/mL. The cultures were incubated at 25 °C for 72 h with 150 rpm of rotary shaking. After 48 and 72 h of incubation, samples were collected and centrifuged at 10,000 g for 10 min. The supernatants obtained were sterilised via filtration through 0.45 µM and stored at −80 °C until use.

The supernatants were mixed individually at 50% (*v*/*v*) with SM agar (2X) at 50 °C and poured into a Petri dish. Once solidified, they were inoculated in the centre with 5 µL of the spore solution of the target mould. Unsupplemented SM agar plates (1X) inoculated with the target mould were used as a control. After inoculation, the SM agar plates were incubated at 25 °C for 7 days. The diameter of the mycelia of each replicate was measured daily in two directions at right angles to each other. The percentage of inhibition of the radial growth of the target mould was calculated as follows:

Inhibition (%) = (DC − DT)/(DC) × 100

where DC is the diameter of the mycelia on the control (mm) and DT is the diameter of the mycelia of the target mould confronted with the supernatant.

##### Production of Lytic Enzymes

Four different enzymatic activities related to the degradation of cell walls were tested. Chitinase and β-glucanase activities were determined as previously described according to Cordero-Bueso et al. [37], via the inoculation of 5 μL of the yeast suspension into specific agar media. Pectinase activity was determined in YPD agar supplemented with 10% apple pectin (Sigma, St. Louis, MO, USA), as previously described by Cabañas et al. [33]. Finally, the protease activity was evaluated in SM agar. Five μL of the yeast suspension was placed onto SM agar and incubated at 15 °C and 25 °C for five days. A clear zone around the colony was considered positive activity and was also measured to assess the intensity of the activity. The determinations were performed in triplicate.

#### 2.5.3. Parasitism of Hyphae

The parasitism interaction between selected yeasts and target moulds was evaluated on SM agar at 2.5% (*w*/*v*) of skim milk powder (8 mL per plate) according to Cabañas et al. [33].

#### 2.5.4. Effect of Iron and Manganese Concentration on the Antagonist Activity

The influence of the iron (Fe) and manganese (Mn) concentration on yeasts producing the inhibitory halo was investigated as described according to Cordero-Bueso et al. [37]. The assay was performed in triplicate on four different media: YPD agar (Condalab), nutrient agar (Condalab), yeast malt agar (YMC; yeast extract 3 g/L, malt extract 3 g/L, peptone 5 g/L, glucose 10 g/L and agar 15 g/L; Condalab), and SM agar at pH 5.5. The culture media were supplemented with 0, 5, 10, and 20 μg/mL of FeCl3 or with 0.1, 1, 6, and 10 μg/mL of manganese ion to investigate the effect of Fe and Mn depletion on the antifungal activity. After the medium solidified, 100 μL of spore suspension from the target mould was spread on plates. After drying, 5 μL of yeast cell solutions was placed on agar plates in equidistant positions. The plates were incubated at 25 °C for 7 days. Then, the size of the inhibition halos was recorded and compared to evaluate the role of competition for Fe or Mn on the antifungal activity of the yeasts.

#### 2.5.5. Production of Antifungal Volatile Organic Compounds (VOCs)

The production of antifungal VOCs by selected yeasts was carried out using a double-dishes system (DDS), as previously described by Ruiz-Moyano et al., with modifications [38]. Briefly, 5 μL of the spore suspension was inoculated at the central point of acidified PDA agar, and it was placed down in the DDS. After that, 100 μL of yeast suspension was spread out on SM agar at pH 5.5, and it was placed on the top of the DDS. Agar plates were confronted and sealed with parafilm™. After that, 4 holes (3 mm × 5 mm) were made in the union of both plates. SM agar at pH 5.5, without yeast inoculation, which was confronted with acidified PDA inoculated with the target mould, was used as a control. The DDS was incubated at 25 °C for 7 days. The experiment was performed in triplicate, with the diameter of each replicate’s mycelium measured daily in two perpendicular directions. The percentage of inhibition of the radial growth of the target mould was calculated as follows:

Inhibition (%) = (DC − DT)/(DC) × 100

where DC is the diameter of the mycelia on the control (mm) and DT is the diameter of the mycelia of the target mould confronted with yeast in the DDS.

The analysis and identification of the volatile compounds produced by yeasts that showed mould inhibition in the DDS and three yeasts without activity (*P. jadinii* PJ173 and PJ1468 and *K. lactis* KL874) were determined 3 and 7 days after the confrontation with gas chromatography/mass spectrometry, as described according to Ruiz-Moyano et al. [38]. The analysis was carried out in an Agilent 6890 GC-5973 MS system (Agilent Technologies, Little Falls, DE, USA) equipped with a 5% phenyl-95% polydimethylsiloxane column (30 m × 0.32 mm inner diameter, 1.05 μm film thickness, Hewlett-Packard, Palo Alto, CA, USA). To detect the potential VOCs, the volatile compounds produced by yeast without activity and control were subtracted from the volatile profile of yeasts with antifungal capacity by VOCs.

#### 2.5.6. Effect of Spore Germination on Spoilage Moulds

One hundred microliters of yeast solution was added to a Petri dish. Subsequently, SM agar made with 2.5% (*w*/*v*) skimmed milk powder and a pH adjusted to pH 5.5 was poured into the dish at approximately 50 °C. Once the medium had solidified, 100 μL of a spore suspension from the target mould was spread out on the SM agar. SM agar without yeast inoculation was used as a control. To assess the impact on germination, a total of 200 spores per plate were observed under a Leica DM 2000 LED microscope (Leica Microsystems SLU, L’Hospitalet de Llobregat, Spain). A spore was considered germinated when the length of the germ tube was equal to or exceeded the spore diameter. The experiment was performed in triplicate.

The inhibition of spore germination was calculated by comparing the germination of the target mould in the presence and absence of yeast (control) using the following equation:

Inhibition of spore germination (%) = (GC − GY)/(GC) × 100

where GC is the number of spores germinated on the control, and GY is the number of spores germinated in the presence of specific yeast.

### 2.6. Evaluation of Antagonist Activity In Vivo

The antagonist activity of the selected yeasts, based on in vitro assays, was investigated on commercial wedges of semi-soft cheese. First, a small incision of approximately 3 mm depth × 3 mm diameter was made in the cheese wedges. This was inoculated with 5 µL of spore suspension of *P. commune*, *M. plumbeus/racemosus*, or *F. verticillioides*. Subsequently, 5 μL of yeast cell solution was added to the same incision. As a control, the yeast solution inoculation was replaced with sterile water. In addition, a positive control was performed with 5 µL of natamycin solution at 1000 mg/L instead of the yeast suspension. Inoculated cheese wedges were placed in Petri dishes and incubated at 25 °C for 12 days. Five replicates were made for each combination, with the diameter of each replicate’s mycelium measured daily in two perpendicular directions. The percentage inhibition of the radial growth of the target mould was calculated as follows:

Inhibition (%) = (DC − DT)/(DC) × 100

where DC is the diameter of the mycelia on the control (mm), and DT is the diameter of the mycelia of the target mould confronted with yeast or natamycin.

### 2.7. Statistical Analysis

The screening of the antifungal activity of yeast and the effect of competition for nutrients (iron and manganese) was evaluated using ‘ward.D2’ hierarchical clustering and plotted with the *pheatmap* package version 1.0.12 [39].

To model the impact of pH, a_w_, and temperature variables (Supplemental Table S2) on inhibition halos, a 3-factor, 3-level Box–Behnken design combined with a surface response methodology was applied. The surface response methodology employed StatGraphics Centurion XVI version 8.0 software. The quadratic model was as follows:

$$Y\;=\;\beta_{0}\;+\;\beta_{1}X_{1}\;+\;\beta_{2}X_{2}\;+\;\beta_{3}X_{3}\;+\;\beta_{12}X_{1}X_{2}\;+\;\beta_{13}X_{1}X_{3}\;+\;\beta_{23}X_{2}X_{3}\;+\;\beta_{11}X_{1}^{2}\;+\;\beta_{22}X_{2}^{2}\;+\;\beta_{33}X_{3}^{2}$$

where *Y* is the response variable (inhibition halo) predicted by the model; *β*_0_ is an offset value; *β*_1_, *β*_2_, and *β*_3_ are the regression coefficients for the main (linear) terms; *β*_11_, *β*_22_, and *β*_33_ are quadratic effects; *β*_12_, *β*_13_, and *β*_23_ are interaction effects; and *X*_1_, *X*_2_, and *X*_3_ are the independent variables. The model was used to estimate the inhibition halo size of each selected yeast strain against the target mould in physicochemical and ripening conditions commonly found in traditional cheeses (Supplemental Table S2). The software also generated an ANOVA, establishing statistical significance at the 95% confidence level. The optimal levels for the antifungal activity (inhibition halo) of each variable analysed were also obtained with the same statistical programme.

Finally, statistical analysis of the data from the inhibition of spore germination, biofilm formation, the inhibition of radial growth by VOCs, and the inhibition of target mould on cheese wedges was carried out using SPSS for Windows, version 21.0 (IBM Corp., Armonk, NY, USA). The differences between groups were studied via one-way ANOVA and separated by Tukey’s honest significant differences test (*p* ≤ 0.05). Moreover, the relationship between the VOCs produced by the selected yeasts and the antifungal activity by this mechanism was established using Pearson correlation coefficients and principal component analysis (PCA) using SPSS software.

## 3. Results

### 3.1. Mould Identification and Screening of Yeast Antagonist Activity

A total of 25 mould isolates were obtained from cheese rinds. They belonged to three different species. The most common species was *P. commune*, with 12 isolates, followed by *M. plumbeus/racemosus*, with 8 isolates, and *F. verticillioides*, with 5 isolates. One isolate from each species was randomly selected as the target for further antifungal evaluation.

**Figure 1:**
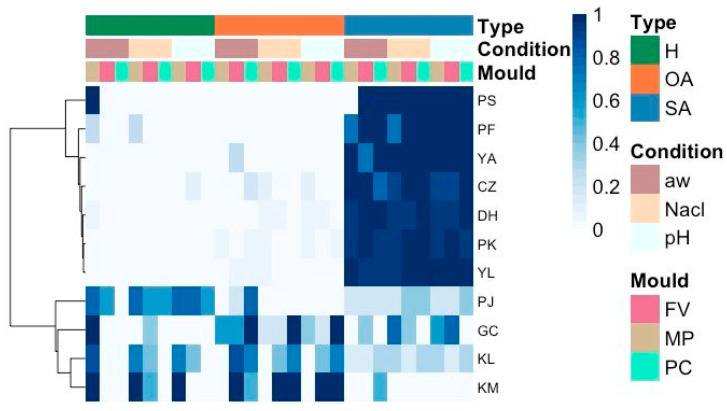
Heatmap presents the yeast species and their antagonist activities in the confrontation assays. The data used in the heatmap is the relative strength of the types of activities. Both columns and rows are grouped using Euclidean distance and average linkage. The unit variance scale is applied to the rows. The colour scale represents the scale of relative strength of antagonist activity of each yeast species (PS, *Pichia sporocuriosa*; PF, *Pichia fermentans*; YA, *Yarrowia alimentaria*; CZ, *Candida zeylanoides*; DH, *Debaryomyces hansenii*; PK, *Pichia kudriavzevii*; YL, *Yarrowia lipolytica*; PJ, *Pichia jadinii*; GC, *Geotrichum candidum*; KL, *Kluyveromyces lactis*; and KM, *Kluyveromyces marxianus*), with dark blue indicating high abundance and white, no abundance. Types of yeast activity were labelled in dark green for inhibition halo, orange for other activities (competence for the space, change in morphology, or intense sporulation), and blue for no activity. The different physicochemical conditions tested were labelled in brown for water activity, beige for NaCl concentration, and light blue for pH results. Target moulds used were labelled in pink for *F. verticillioides*, brown for *M. plumbeus/racemosus*, and green for *P. commune*. (For interpretation of the references to colour in this figure legend, the reader is referred to the Web version of this article.).

A set of 84 yeast isolates belonging to 11 different species native to traditional soft raw ewe’s milk cheeses was confronted with the three target moulds in two different media, the PDA and SM agar. No activity was observed on PDA agar, while various types of interactions were observed on SM agar. The antagonist yeast activities on SM agar were classified into three groups: halo activity (H), other activities (OA: competition for space, morphology changes, and intense sporulation around the colony), and no appreciable activity (SA). Figure 1 shows a heatmap scaled by yeast species (row) representing the influence of the different conditions tested (water activity, %NaCl and pH) on the antagonist yeast activity against the three target moulds. Cluster analysis shows that the yeasts fall into two main clusters: one group with species that showed low or no activity, and another group with the most interesting species that showed stronger activity. In the first group, *Pichia sporocuriosa* was highlighted, which produced a halo against *M. plumbeus/racemosus* under certain water activity conditions. At a lower intensity, some strains of *Pichia fermentans* produced an inhibition halo against the same target mould under certain a_w_ and NaCl concentration conditions. The rest of the yeast species within this cluster did not show any relevant activity. In contrast, the other large cluster contains the species that showed stronger antagonist activity. Four species stand out in this cluster: *Pichia jadinii*, *Geotrichum candidum*, *K. lactis*, and *K. marxianus*. Most of the isolates of these species showed intense activity against *M. plumbeus/racemosus* and *F. verticillioides*, while weak or null activity was observed against *P. commune*. Regarding the type of activity, *P. jadinii* and *G. candidum* isolates developed mainly inhibition halos and competition for space, respectively. However, in the case of *Kluyveromyces* species (*K. lactis* and *K. marxianus*), they showed different types of activity, such as inhibition halos, morphology changes, and intense sporulation around the colony. Finally, regarding the influence of physicochemical conditions on the antagonist activity, the a_w_ was the factor that showed the strongest influence, while less variability was observed between the pH and NaCl conditions tested. At a lower a_w_, the species *G. candidum* displayed higher antifungal activity via the inhibition halo and competition for the space against *M. plumbeus/racemosus*. In contrast, *Kluyveromyces* activity via the halo against *F. verticillioides* was limited at a lower a_w_.

Based on these results, we selected 15 yeast strains with the strongest inhibition halo formation and competition for the space for further analysis (*G. candidum*: GC663; *P. jadinii*: PJ173, PJ433, PJ659, PJ1008, and PJ1468; *K. lactis*: KL371, KL874, KL890, KL904, KL1098, KL1351, and KL1507; *K. marxianus*: KM364 and KM1070).

### 3.2. Impact of Cheese Ripening Conditions on the Antagonist Activity

To assess the combined impact of three key ripening factors (temperature, a_w_, and pH) on the antagonist activity of selected yeasts, a BBD experimental design was performed. Supplementary Table S3 presents the mean, standard deviation, maximum, and minimum values of the radius of the inhibition halo caused by the selected yeasts against the growth of *M. plumbeus/racemosus* and *F. verticillioides*. *P. commune* was not included in this assay as no yeast showed relevant antagonist activity against it. Moreover, *G. candidum* GC663 was excluded due to the inherent challenges in quantifying its antagonistic activity, which is predominantly mediated through spatial competition. In general, *P. jadinii strains* (PJ173, PJ433, PJ659, and PJ1008) exhibited the largest inhibition halos against *F. verticillioides* and *M. plumbeus/racemosus*, with mean values of around 2.5 and 3.1 mm, respectively. Conversely, a lower activity was observed in isolates of *K. lactis* and *K. marxianus* species, with mean inhibition halos of 1.86 and 0.85 mm, respectively, against both target moulds.

Table 1 presents the statistical analysis of the quadratic response surface model and the optimal cheese ripening conditions for the antifungal activity of selected yeast strains against *F. verticillioides*. Out of the fourteen yeast strains tested, seven (four *P. jadinii* and three *Kluyveromyces*) showed a good fit with the model’s factors and ranges (adjusted R^2^ > 50%). For *P. jadinii*, temperature emerged as the most significant factor in the model for four of the five strains, with both linear (A) and quadratic (AA) terms being significant (*p* ≤ 0.05). Specifically, the antifungal activity model for strain PJ173 exhibited an adjusted R^2^ value of 95.59, with the optimal inhibition of *F. verticillioides* at 4.4 mm under conditions of 16.6 °C, a_w_ of 0.87, and pH 5. Similar values were observed for the other *P. jadinii* strains. For the *Kluyveromyces* strains, the models indicated a limited influence of the tested factors and their interactions within the range of values representative of cheese ripening conditions. Consequently, most of these models were excluded from the analysis due to adjusted R^2^ values below 50%, reflecting a poor model fit. However, in the models analysed for some *Kluyveromyces* strains (adjusted R^2^ values > 50%), the optimal inhibition values ranged between 8 and 8.4 °C, 0.84 and 0.87 aw, and pH from 4.5 to 5.5. For strain KM1070 with an adjusted R^2^ value of 62.11, an inhibition zone of 3.92 mm was estimated at a temperature of 8.4 °C, aw of 0.84, and pH 4.5. The rest of the strains had lower antifungal activity under similar conditions.

Regarding the impact of factors on the ability to inhibit the growth of *M. plumbeus/racemosus*, the activity of 8 out of the 14 strains studied (5 *P. jadinii* and 3 *Kluyveromyces*) was well adjusted to the model (adjusted R^2^ > 50; Table 2). Once again, temperature emerged as the most significant factor, along with a_w_. For *P. jadinii* strains, PJ173 exhibited a higher estimated inhibition value (5.87 mm) at 15.5 °C, 0.97 a_w_, and pH 4.5. Slightly lower estimated inhibitions were observed for the other *P. jadinii* strains at a similar temperature. In the case of *Kluyveromyces* strains, KM10170 exhibited the best estimated inhibition with 4.42 mm at 8 °C, 0.84 a_w_, and pH 4.8. The other strains, well adjusted to the model, had lower estimated activity under their optimal conditions, around 11 °C, and variable a_w_ and pH. Notably, strain *K. lactis* KL371’s activity was significantly influenced by a_w_, with an optimum at 0.84.

### 3.3. Characterisation of the Mechanism of Antagonist Activity

Table 3 presents the results for antibiosis, biofilm formation, lytic enzyme production, and parasitism. Regarding the antibiosis capacity of the selected yeasts, none of the supernatants significantly reduced the growth of the spoilage target moulds. Similarly, most of the selected yeasts exhibited a poor ability to form biofilms in vitro. Only *K. marxianus* KM1070 demonstrated a moderate ability to adhere to the bottom of polystyrene wells compared to the positive control. In contrast, different lytic enzymatic activities, including pectinase, β-glucanase, chitinase, and protease, were observed. In general, all strains of the same species showed similar enzymatic abilities. The strain *G. candidum* GC663 stood out for its pectinase and β-glucanase activities. *K. lactis* species were characterised by protease activity at 15 °C and to a lesser extent at 25 °C. The intensity of the strain *K. lactis* KL1507 stands out among the selected strains of this species. On the other hand, no lytic enzyme activities were detected in the other *Kluyveromyces* species, *K. marxianus*. Finally, the enzymatic activities exhibited by the *P. jadinii* strains were variable among the strains evaluated. All strains of this species showed β-glucanase activity at different levels. Interestingly, *P. jadinii* strain PJ1008 also showed pectinase activity, and *P. jadinii* strains PJ433 and PJ1468 showed protease activity. This activity is closely related to the parasitic ability of the hyphae. Specifically, 8 of the 15 selected yeasts showed no adhesion to the hyphae of the target moulds. The remaining strains exhibited variable parasitic ability. Among them, *P. jadinii* PJ433, which demonstrated parasitism against *P. commune* and *F. verticillioides*, and *G. candidum* GC663 against all three target moulds tested, stood out (Figure S1).

**Figure 2:**
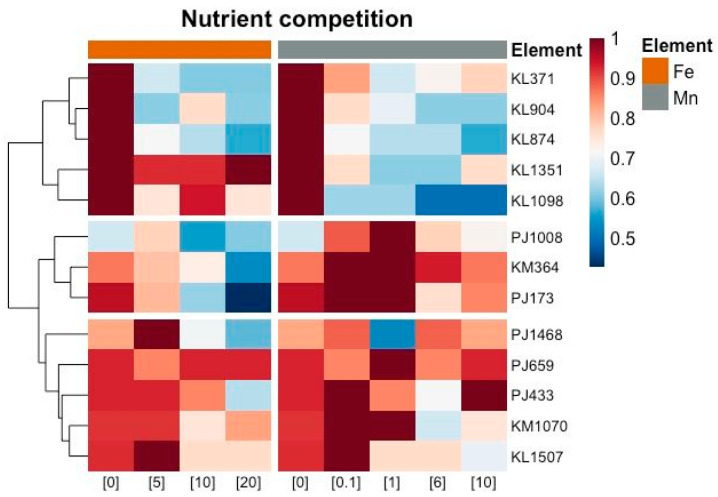
Heatmap of the ability to compete for iron (orange) and manganese (grey) of the different yeasts selected by inhibition halo at the concentrations reflected on the lower axis (in mg/mL) against *Mucor plumbeus/racemosus*. The data used in the heat map are the relative size of the different halos produced. Both columns and rows are grouped using Euclidean distance and mean linkage. The unit variance scale is applied to the rows. The colour scale represents the relative abundance scale of the halo intensity of the different strains (PJ, *Pichia jadinii*; GC, *Geotrichum candidum*; KL, *Kluyveromyces lactis*; KM, *Kluyveromyces marxianus*), with dark red indicating high abundance and dark blue, no abundance.

Another parameter studied was the competition for nutrients, iron, and manganese. Figure 2 shows a heatmap scaled by selected yeast strains that produced an inhibition halo (row) representing the influence of different nutrient concentrations tested on antifungal yeast activity against the selected target mould (*M. plumbeus/racemosus*). The results revealed two major clusters of yeasts: one consisting of strains that exhibited antagonistic activity due to nutrient competition, and the other consisting of strains that did not exhibit this activity. Within the first cluster, the five strains (KL371, KL904, KL874, KL1351, and KL1098) all belong to the same species, *K. lactis*. These strains showed activity in the confrontation control without the addition of nutrients, but for four of the five strains, the activity decreased as the concentration of both nutrients increased. These results indicate that competition for Fe and Mn is relevant in the antagonist activity of these strains. The exception was strain KL1351, which showed mould inhibition with an increasing Fe concentration, indicating that competition for this strain was only for Mn. The second cluster included yeasts for which nutrient competition was less important in their inhibition mechanism. Within this cluster, there were two subgroups: one consisting of strains that competed only for Fe, including *P. jadinii* PJ1008 and PJ173, and *K. marxianus* KM364; and the second subgroup consisting of five strains (*P. jadinii* PJ433, PJ659, and PJ1468, *K. lactis* KL1597, and *K. marxianus* KM1070) that did not compete for any of the nutrients, characterised by their antagonistic activity at different levels of nutrient concentration. Therefore, the mode of control of mould growth by these yeasts is not dependent on nutrient competition.

**Figure 3:**
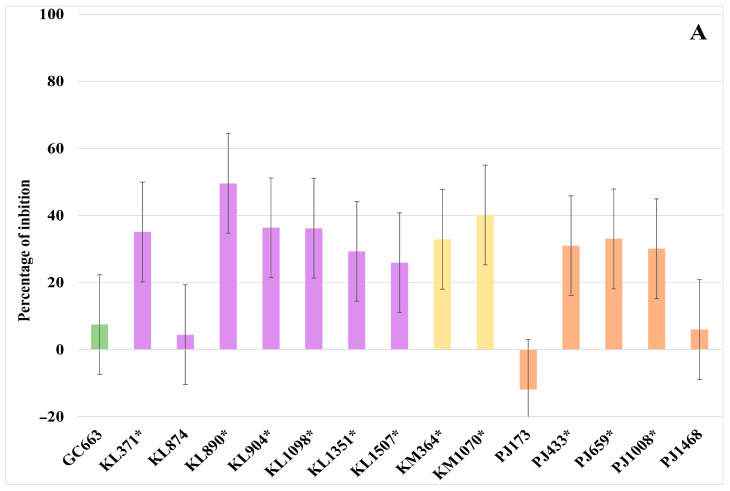

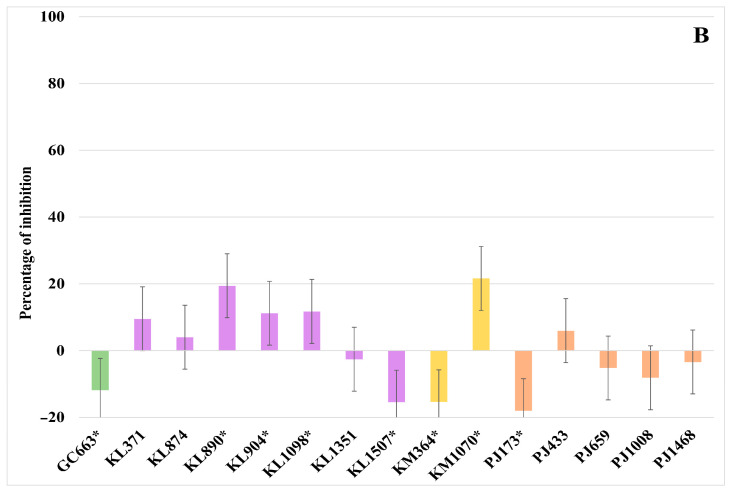
Growth inhibition percentage of *Fusarium verticillioides* (**A**) and *Mucor plumbeus/racemosus* (**B**) when exposed to volatile organic compounds (VOCs) produced by the selected yeasts. On the ordinate axis, positive values mean reduced growth, negative values mean increased mycelial growth. Species are distinguished by colour: green, *Geotrichum candidum*; lavender, *Kluyveromyces lactis*; yellow, *Kluyveromyces marxianus*; and orange, *Pichia jadinii*. * Indicates yeasts with significant activity (*p* ≤ 0.05). Error bars correspond to the 95% confidence interval from Tukey’s honestly significant difference test ((**A**): ±14.87; (**B**): ±17.89).

Regarding the inhibitory capacity mediated by VOCs, Figure 3 presents the yeast strains that significantly (*p* ≤ 0.05) reduced the radial growth of the target moulds. Generally, the antagonist activity of VOCs was strain-dependent and exhibited substantial variability across the tested moulds. None of the yeasts significantly inhibited the growth of *P. commune*. In fact, 14 out of the 15 yeast strains promoted the growth of this mould to varying degrees depending on the specific strain (Figure S2). However, four strains—three K. lactis (KL890, KL904, and KL1098) and one *K. marxianus* (KM1070)—significantly (*p* ≤ 0.05) decreased the growth of the other two target moulds, *M. plumbeus/racemosus* and *F. verticillioides*. Among these, strains KL890 and KM1070 demonstrated the most pronounced effects. Specifically, both strains reduced the growth of *M. plumbeus/racemosus* by approximately 30%, while the KL890 strain exhibited the highest activity against *F. verticillioides* (49.6%), followed by KM1070 (40%). Furthermore, nine additional strains—five *K. lactis* (KL371, KL904, KL1098, KL1351, and KL1507), one *K. marxianus* (KM369), and three *P. jadinii* (PJ433, PJ659, and PJ1008)—showed the significant *(p* ≤ 0.05) inhibition of *F. verticillioides* growth, ranging from 22% to 38%. In contrast, four strains (GC663, KL874, PJ173, and PJ468) exhibited limited activity, with mean growth reduction values below 10%.

**Figure 4:**
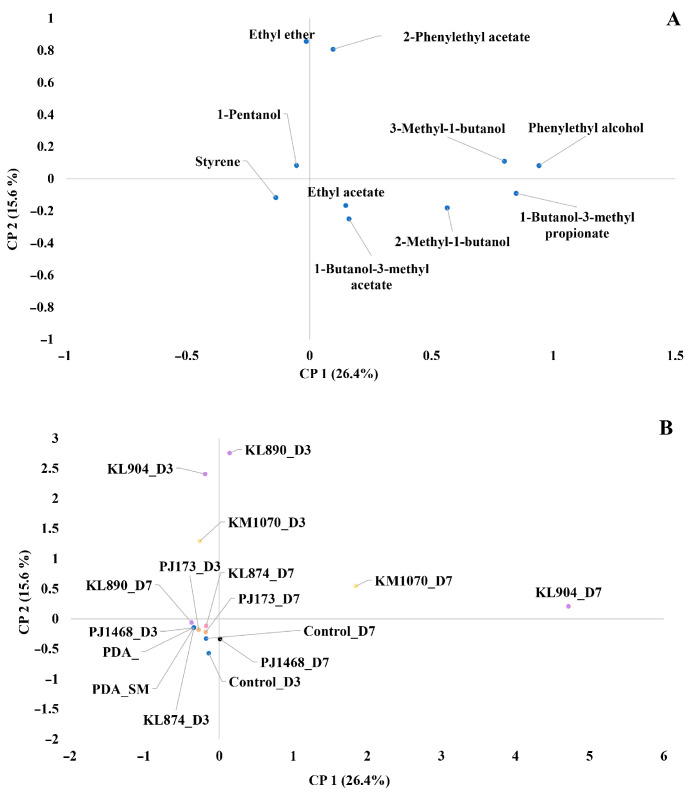
Projection of samples grouped according to the main volatile organic compounds identified (**A**) and yeast strain treatment into the double dish systems confrontation against *Fusarium verticillioides* at 3 (D3) and 7 (D7) days (**B**) in the space defined by the first two components (PC1: 26.4% of variability/PC2. 15.6% of variability): *Kluyveromyces lactis* KL890 (KL890_3 and KL890_7), *Kluyveromyces lactis* KL904 (KL904_D3 and KL904_D7), *Kluyveromyces lactis* KL874 (KL874_D3 and KL874_D7), *Kluyveromyces marxianus* KM1070 (KM1070_D3 and KM1070_D7), *Pichia jadinii* PJ173 (PJ173_D3 and PJ173_D7), and *Pichia jadinii* PJ1468 (PJ1468_D3 and PJ1468_D7). PDA_SM, PDA, ControlD3, and ControlD7 acronyms represent control treatments of the double dish system of PDA and SM agar without target mould, PDA agar without target mould, target mould inoculated in the double dish system at 3 days, and target mould inoculated in double dish system at 7 days.

The potential VOCs responsible for this activity against the target mould *F. verticillioides* were identified and characterised using GC-MS. For this purpose, six yeast strains were selected: the three yeasts with the highest antagonist activity by this mechanism (KL890, KL904, and KM1070), as well as three yeasts without activity (PJ173, PJ1468, and KL874). Figure 4 shows the PCA loading and score plots of the volatile compounds detected and yeast treatments. The analysis of principal component 1 (26.4% of variability) and principal component 2 (15.6% of variability) revealed that PC1 was defined on its positive plane by strains *K. marxianus* KM1070 and *K. lactis* KL904 after 7 days of confrontation, and VOC-mediated inhibition was associated with the production of phenylethyl alcohol and 1-butanol-3-methyl propionate. Principal component 2 was defined on its positive plane by strains *K. lactis* KL890 and KL904 after 3 days of confrontation, and its activity was associated with the presence of ethyl ether and 2-phenylethyl acetate.

**Figure 5:**
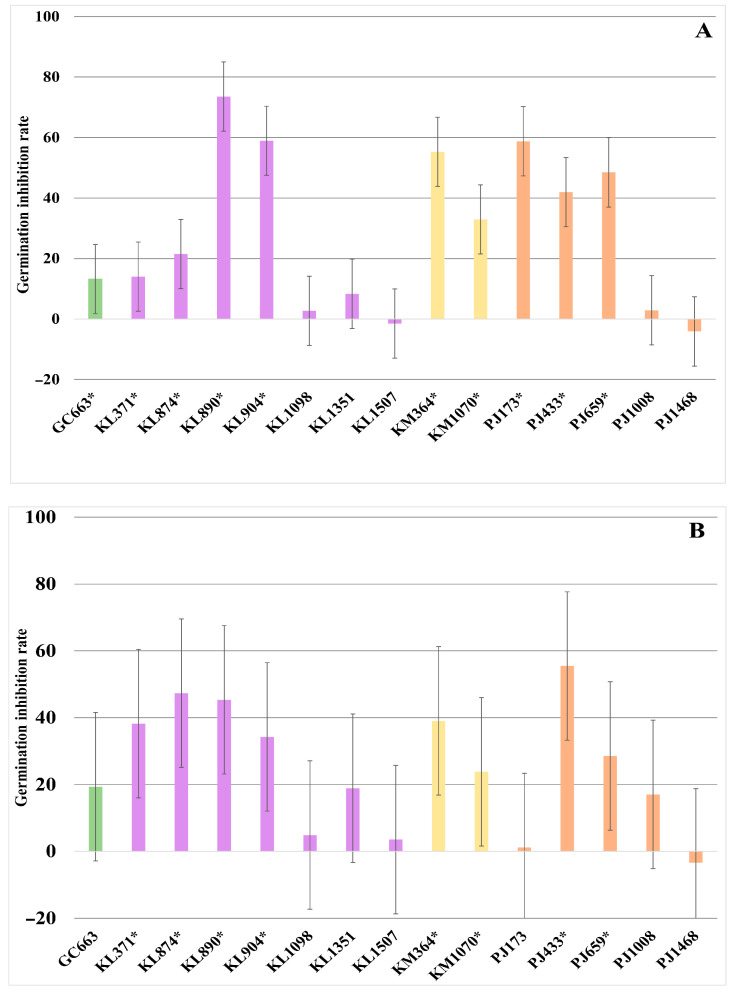
Spore germination inhibition percentage of *Fusarium verticillioides* (**A**) and *Mucor plumbeus/racemosus* (**B**) when exposed to confrontation with the selected yeasts. On the ordinate axis, positive values mean reduced germination, and negative values mean increased germination. Species are distinguished by colour: green, *Geotrichum candidum*; lavender, *Kluyveromyces lactis*; yellow, *Kluyveromyces marxianus*; and orange, *Pichia jadinii*. * Indicates yeasts with significant activity (*p* ≤ 0.05). Error bars correspond to the 95% confidence interval from Tukey’s honestly significant difference test ((**A**): ±11.44; (**B**): ±22.21).

Finally, the inhibitory effect of selected yeasts on spore germination was evaluated. The three target moulds exhibited varying sensitivity, with *P. commune* demonstrating the highest resistance. The spore germination of this species was not significantly inhibited by any of the yeasts (*p* > 0.05) (Figure S3). Consistent with VOC activity, several yeast strains promoted *P. commune* spore germination. However, the spore germination of *M. plumbeus/racemosus* and *F. verticillioides* was inhibited to varying degrees by the selected strains (Figure 5). Overall, 10 of the 15 selected yeasts significantly (*p* ≤ 0.05) inhibited the spore germination of these two moulds. Specifically, six yeast strains inhibited the *F. verticillioides* spore germination by exceeding 40%. Among these, strains *K. marxianus* KM364, *P. jadinii* PJ173, and *K. lactis* KL904 and KL890 exhibited inhibition rates of 55.3%, 58.8%, 58.9%, and 73.5%, respectively (Figure 5A). In contrast, the inhibition of *M. plumbeus/racemosus* spore germination was generally lower than that observed for *F. verticillioides*. Although it is worth noting that three strains, *K. lactis* KL890 and KL874 and *P. jadinii* PJ433, still exhibited notable inhibition rates of 45.3%, 47.3%, and 55.5%, respectively (Figure 5B).

### 3.4. Antagonist Activity In Vivo on Cheese Wedge

**Figure 6:**
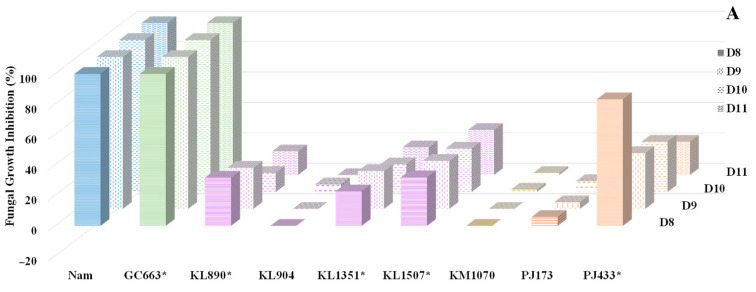

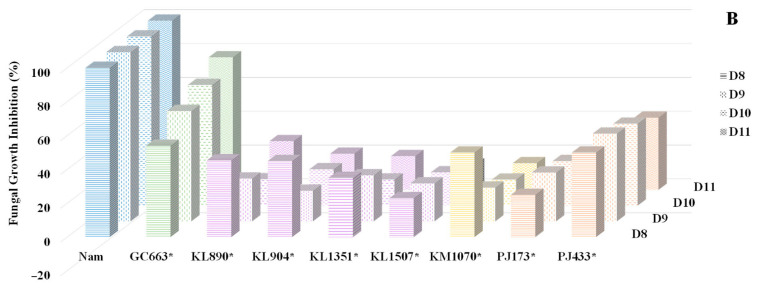
Growth inhibition percentage of *Fusarium verticillioides* (**A**) and *Mucor plumbeus/racemosus* (**B**) when exposed to eight yeast strains during the 11 days of confrontation on soft cheese wedges. Species are distinguished by colour: in blue, the positive controls, Nam = natamycin (1000 mg/L); in green, *Geotrichum candidum*; in lavender, *Kluyveromyces lactis*; in yellow, *Kluyveromyces marxianus*; and orange, *Pichia jadinii*. The different growth days are shown with various textures. * Indicates the yeast strains that produced significant inhibition (*p* ≤ 0.05) in at least 2 storage days. The 95% confidence interval from Tukey’s honestly significant difference test was (**A**): ±15.15; (**B**): ±12.69.

Based on the prior characterisation of antagonist activity, eight yeast strains were selected for in vivo evaluation within a cheese matrix against three target moulds through confrontation assays. As a positive control, natamycin consistently demonstrated the complete inhibition of all target moulds. The results confirmed that the antagonist activity was strain dependent. Regarding the activity against *P. commune*, it exhibited significant resistance to the antagonist, with only *K. lactis* KL904 and *P. jadinii* PJ433 achieving modest growth inhibitions of 21% and 18%, respectively, after 11 days of storage (Figure S4). For the other target moulds, Figure 6A,B present the percentage inhibition of *F. verticillioides* and *M. plumbeus/racemosus* by the selected yeast strains over the 11-day storage period. *G. candidum* GC663 demonstrated notable efficacy by significantly limiting their growth by 54–78% and 100%, respectively, confirming its ability to compete for space (Figure S5). *P. jadinii* PJ433 exhibited moderate activity against these moulds, with inhibition rates of approximately 30% and 45% against *M. plumbeus/racemosus* and *F. verticillioides*, respectively, after 9 days (Figure S6). Conversely, strains belonging to the genus *Kluyveromyces* generally showed lower antifungal activity. Three *Kluyveromyces* yeasts (*K. lactis* KL890, KL1351, and KL1507) significantly (*p* < 0.05) reduced *F. verticillioides* growth by 15–32% (Figure 6A). Their antifungal activity was more pronounced against *M. plumbeus/racemosus*, with four of the five *Kluyveromyces* strains significantly (*p* ≤ 0.05) reducing its growth by 35–50% after eight days of storage. However, this activity declined to below 28% as the trial progressed (Figure 6B).

## 4. Discussion

Mould contamination in cheese is a critical concern, as it directly impacts product quality and poses a food safety risk, resulting in considerable economic losses. To address this, the dairy industry has focused on mitigating its reliance on chemical preservatives, instead embracing contemporary strategies for producing more natural and consumer-friendly products. This study therefore characterised the antagonist capacity of cheese-native yeasts against three spoilage mould species in this product. We first identified the main moulds contaminating raw ewe’s milk soft cheese, finding three predominant species: *P. commune*, *M. plumbeus/racemosus,* and *F. verticillioides*. *P. commune* and *M. plumbeus/racemosus* are among the dominating spoilage moulds in artisanal cheeses. Furthermore, *P. commune* is one of the major producers of CPA within the *Penicillium* genus [2]. Less common in cheese is the presence of *F. verticillioides.* This species is predominantly reported as a mycotoxin-producing pathogen on maize [40]; however, it has also been identified as a contaminating mould on artisanal cheese with a higher water content [41,42].

The use of direct plate confrontation is a highly effective and technically straightforward method for evaluating the antagonistic potential of microorganisms, enabling the rapid screening of numerous isolates [43]. Using this technique, 15 out of 84 yeast strains, encompassing *G. candidum*, *K. lactis*, *K. marxianus*, and *P. jadinii*, exhibited notable antagonist activity on SM agar against *M. plumbeus/racemosus* and *F. verticillioides*. To the best of our knowledge, these yeast species have not been previously reported to limit the growth of these specific cheese spoilage moulds. It should be noted, however, that *P. jadinii* and *Kluyveromyces* spp. have demonstrated antifungal activity against toxigenic moulds belonging to the genera *Aspergillus*, *Penicillium*, and *Fusarium* [23,24,44,45,46]. *P. commune* exhibited enhanced resistance to antagonist yeast compared to the other species tested. Specific studies investigating the resistance of *P. commune* to biocontrol agents are limited. However, consistent with the present findings, *P. commune* has demonstrated greater resistance to LAB biocontrol agents compared to other *Penicillium* species [47,48]. In contrast, previous studies have reported variable susceptibility patterns concerning *Mucor* species [49,50]. These studies suggested that the susceptibility of target moulds to biocontrol agents is influenced by the antagonist mechanisms or the specific modes of action of the evaluated yeast strains. Moreover, the *D. hansenii* strains examined in this study did not exhibit significant antagonist activity, a finding that contrasts with numerous previous reports detailing substantial antifungal properties in other strains of this species [21,22,51]. The antagonist activity was indeed significantly influenced by the composition of the nutrient medium. None of the strains exhibited antagonist activity on PDA agar under the same conditions. This finding is consistent with scientific works focused on the selection of biocontrol antifungal agents [16,33], since the metabolic activity of microorganisms is strongly influenced by environmental conditions such as nutrient composition. SM agar, contrary to PDA agar, is rich in proteins (caseins), which are precursors of antifungal compounds such as fatty acids, peptides, organic acids, and VOCs [52,53].

The selection of biocontrol agents requires rigorous evaluation of their antagonistic capacity in the environmental conditions of the target ecological niche [54]. In this context, candidate strains must exhibit robust adaptation and an adequate physiological response to the key abiotic stresses associated with cheese ripening, such as temperature, pH, and a_w_. Ripening in most artisan cheeses typically occurs at 4–15 °C, with pH values generally between 4.5 and 6.0 and a_w_ above 0.92 [35,36]. To understand the impact of these parameters, we modelled the behaviour of strains capable of producing an inhibitory halo against target moulds. In general, *P. jadinii* showed higher estimated antifungal activity than the *Kluyveromyces* strains. The factors studied showed a limited influence on *Kluyveromyces* activity, although temperature notably affected most *P. jadinii* strains. The model demonstrated a strong fit for most *P. jadinii* strains, enabling the identification of optimal activity against *M. plumbeus/racemosus* and *F. verticillioides* at around 16 °C. In contrast, *Kluyveromyces* strains showed more variable responses; those accurately described by the model exhibited optimal activity within the 8–11 °C temperature range and were only sporadically influenced by the a_w_. These findings suggest that the selected strains can exert notable antagonist activity during the ripening phase of most cheese varieties. In addition, the species- and strain-specific responses to ripening factors indicate that protective cultures can be strategically tailored to align with the distinct physicochemical dynamics of individual cheese varieties.

Hence, a comprehensive understanding of the mechanisms of action of biocontrol agents is essential for optimising their formulation and application strategies. Elucidating the mechanism of action underlying the antagonist activity of a microorganism presents a significant challenge, as multiple mechanisms often operate synergistically to exert the observed effects. Yeasts can inhibit mould growth through multiple mechanisms, including nutrient and space competition, mycoparasitism, VOCs, mycocins (killer proteins), antifungal peptides, and lytic enzymes [18]. *G. candidum* GC663 inhibited target moulds’ growth primarily through space competition at salt concentrations below 3%. This antagonistic effect is attributed to the yeast’s faster growth rate compared to the spoilage moulds. The optimal growth conditions for *G. candidum*, combined with its dimorphic characteristic (existing in both yeast and hyphal forms), explain this competitive advantage. Specifically, this species exhibits a mould-like rapid radial growth on SM agar at 25 °C with salt concentrations under 2.5%, thereby preventing the expansion of target moulds [19,55]. This strain also demonstrated mycoparasitism against the hyphae of the three target moulds. Although this antagonistic mechanism is infrequently reported and poorly investigated in yeasts [18], it is hypothesised that lytic enzyme production is crucial for hyphal parasitism, given the composition of mould cell walls (≥20% chitin, 50–60% glucan, 20–30% proteins) [56]. Consistent with this, *G. candidum* GC663 exhibited robust β-glucanase and pectinase activity. However, a distinct profile of hyphal attachment was observed for *P. jadinii* PJ1008, despite its similar hydrolytic enzyme profile. The absence of a clear relationship between the lytic enzyme profile and observed mycoparasitism in this work indicates that this phenomenon is strain-dependent and mould-selective. This finding aligns with previous research on *Metschnikowia pulcherrima* [57], indicating that additional mechanisms, beyond the presence of specific lytic enzymes, are likely involved in successful parasitism.

The characterisation of additional antagonist strains revealed their ability to generate inhibition halos, though the underlying mechanisms responsible for these inhibitory effects varied. In the present study, the specific mechanisms of action varied among *Kluyveromyces* strains. Notably, competition for trace elements, specifically Fe and Mn, was prevalent among most *K. lactis* strains. Additionally, two *P. jadinii* strains (PJ1008 and PJ173) and one *K. marxianus* strain (KM364) competed exclusively for Fe. Both Fe and Mn are essential for the growth of nearly all microorganisms, and competition for these trace elements is a well-established antagonistic mechanism [18,58]. Certain yeast species can synthesise high-affinity iron-chelating siderophores, such as pulcherriminic acid (PA). For instance, Krause et al. [59] identified a PA gene cluster in *K. lactis* that may confer ecological advantages. Conversely, our study represents, to our knowledge, the first documented instance of Mn competition by a potential antagonist yeast. While novel for yeasts, this antagonist mechanism has recently been reported in several *Lactobacillus* species active against dairy spoilage fungi [60]. Interestingly, four of the nine *Kluyveromyces* strains demonstrated significant VOC-mediated inhibition of *M. plumbeus/racemosus* and *F. verticillioides* growth. These findings showed a high correlation with the strains’ capacity to inhibit the germination of both target moulds’ spores, indicating that VOCs are likely a primary mechanism for this antagonism. Previous studies have demonstrated the ability of *Kluyveromyces* yeasts to produce VOCs effective against plant spoilage moulds [46,61]. A comparative analysis of the volatile compounds synthesised by VOC-active and VOC-inactive yeast strains identified phenylethyl alcohol, 1-butanol-3-methyl propionate, ethyl ether, and 2-phenylethyl acetate as potentially mediating this antifungal activity. Furthermore, the production of these specific compounds by antagonistic yeasts within the genera *Hanseniaspora*, *Pichia*, and *Meyerozyma* has been correlated with the inhibition of various mould species, including those belonging to *Botrytis*, *Aspergillus*, *Penicillium*, *Mucor*, and *Fusarium* [38,62,63]. In the case of *P. jadinii* strains, their notable antagonist activity against *M. plumbeus/racemosus* and *F. verticillioides* did not correlate with the antagonistic mechanisms assessed. All strains exhibited β-glucanase activity; however, parasitism appeared strain dependent when targeting these moulds. To date, the literature only reports a single *P. jadinii* strain (Y273) with strong antagonistic potential against toxigenic *Aspergillus* species via VOC production [44,64,65]. In our investigation, however, VOC-mediated antifungal activity was limited, and antibiosis via the secretion of antifungal metabolites was negative across all strains. The production of antifungal metabolites by biocontrol agents is a complex process, as it can involve a diverse array of active molecules, including chemical and proteinaceous compounds, whose synthesis is influenced by various abiotic and biotic factors. Given the observed antifungal activity exhibited by *P. jadinii* strains, future research should prioritise elucidating the primary mechanisms underlying their activity. In this regard, OMICs tools offer significant potential to advance our understanding of this issue [66].

The confrontation assays conducted in the cheese matrix partially corroborated the findings from the in vitro screening and characterisation of antagonistic mechanisms. Strains belonging to the genus *Kluyveromyces* demonstrated comparatively limited antagonist activity. This reduced efficacy may be attributed to the experimental conditions of the in vivo assay, which was performed in unsealed Petri dishes with a relatively large headspace. Such conditions likely restricted the accumulation of VOCs, which we demonstrated to be involved in the antifungal capacity of specific strains. The application of these yeasts, or the VOCs associated with their activity, could be useful in developing protective packaging for the storage stability of the final product. Furthermore, *K. lactis* strains displayed competition for essential micronutrients such as Fe and Mn, which are more concentrated in cheese than milk [67]. This increased nutrient availability may have diminished the competitive advantage of these strains, thereby reducing their antagonistic potential. *G. candidum* GC663 and *P. jadinii* PJ433 exhibited promising potential as biocontrol agents in cheese, although they could not reach natamycin’s effectiveness. Notably, *G. candidum* is a yeast species well recognised for its technological role in cheese ripening, and has been proposed by several authors as an adjunct culture in cheese production. However, its excessive growth on the cheese surface can lead to a defect known as ‘toad skin’ [19]. Similarly, *P. jadinii* PJ433 has recently been suggested for use in cheese ripening applications [29]. Despite their potential, further research is required before these strains can be recommended for commercial use as biocontrol agents. Validation under industrial-scale conditions is essential to assess their effects on the physicochemical, microbiological, and sensory properties of cheese. Moreover, the microbial ecology of ripened cheeses is highly complex, with microbial interactions influencing metabolic activities and, consequently, the efficacy of biocontrol agents. Understanding these dynamics will be critical for the successful integration of these yeasts into cheese production systems.

## 5. Conclusions

Screening for antifungal activity on milk agar identified 15 yeast strains from the species *P. jadinii*, *K. lactis*, *K. marxianus*, and *G. candidum* with inhibitory effects against the cheese-spoilage moulds *M. plumbeus/racemosus* and *F. verticillioides*. However, none of the tested strains exhibited significant activity against *P. commune*. Cheese ripening factors, particularly temperature and, to a lesser extent, aw, were found to significantly influence antagonist activity.

The underlying mechanisms of antagonism varied by species and strain. *K. lactis* was notable for its proteolytic activity and nutrient competition, with two strains (KL890 and KL904) producing antifungal VOCs, including phenylethyl alcohol and 1-butanol-3-methyl propionate. *G. candidum* GC663 demonstrated strong spatial competition, parasitism, and notable pectinase and β-glucanase activity. All *P. jadinii* strains exhibited β-glucanase activity, with strain PJ433 also showing significant proteolytic potential. The relatively large inhibition halos observed for *P. jadinii* strains suggest the involvement of additional, unidentified antifungal mechanisms. Finally, in cheese matrix assays, *G. candidum* GC663 and *P. jadinii* PJ433 showed high and moderate efficacy, respectively, in reducing the growth of F. verticillioides and *M. plumbeus/racemosus*. These findings highlight their potential as natural biocontrol agents. Nonetheless, further research is necessary to elucidate their full spectrum of antifungal mechanisms and to validate their performance under industrial-scale conditions, including their impact on cheese quality.

## Figures and Tables

**Table 1:** ANOVA results for the quadratic response surface model and optimal conditions for inhibiting *F. verticillioides* via the selected yeasts.

	KL371 ^1^	KL874	KL890	KL904	KL1098	KL1351	KL1507	KM364	KM1070	PJ173	PJ433	PJ659	PJ1008	PJ1468
**A: Temperature**	0.071	0.536	0.536	0.232	0.403	0.275	0.314	0.314	0.133	0.000 **	0.030 *	0.006 *	0.016 *	0.312
**B: a_w_**	0.593	0.103	0.045 *	0.748	0.128	0.275	0.314	0.314	0.286	0.076	0.363	0.036 *	0.188	0.438
**C: pH**	0.593	1.000	0.536	0.063	0.403	0.451	0.020 *	0.314	0.133	1.000	1.000	0.147	0.356	0.438
**AA**	0.154	0.030 *	0.193	0.565	0.562	0.224	0.306	0.720	0.278	0.000 **	0.014 *	0.023 *	0.016 *	0.675
**AB**	0.167	1.000	0.391	0.651	1.000	0.144	0.064	0.465	0.020 *	1.000	0.511	1.000	0.504	0.463
**AC**	1.000	0.391	1.000	0.209	0.253	1.000	0.465	0.465	0.052	1.000	0.511	1.000	0.210	0.463
**BB**	0.407	0.283	0.061	0.331	0.027 *	0.443	0.720	0.306	0.278	0.189	0.355	0.407	0.457	0.952
**BC**	0.456	0.391	0.391	0.381	0.049 *	1.000	0.465	0.465	0.152	1.000	0.511	0.456	1.000	0.708
**CC**	0.407	0.089	0.776	0.477	0.562	0.443	0.306	0.306	0.702	0.006 *	0.355	0.057	0.195	0.515
***ANOVA (R*** **^2^** ***statistics)***														
**R^2^**	72.36	79.57	77.86	72.36	82.95	66.91	82.35	62.50	86.47	98.42	84.11	90.60	86.87	49.25
**R^2^ adjusted**	-	-	-	-	52.27	-	50.59	-	62.11	95.59	55.51	73.69	63.22	-
***Optimal inhibition values***														
**A: Temperature**	-	-	-	-	8.0	-	8.0	-	8.4	16.6	15.9	16.9	16.2	-
**B: a_w_**	-	-	-	-	0.87	-	0.84	-	0.84	0.87	0.88	0.84	0.87	-
**C: pH**	-	-	-	-	4.50	-	5.50	-	4.50	5.00	4.98	4.80	5.20	-
**Estimated value**	-	-	-	-	2.97	-	2.88	-	3.92	4.38	4.17	4.03	3.64	-

^1^ The initials belong to the species of the strains: KL (*Kluyveromyces lactis),* KM (*Kluyveromyces marxianus*), and PJ (*Pichia jadinii*). * *p*-values of less than 0.05 are statistically significant (bold type). ** *p*-values of less than 0.001 are statistically significant (bold type).

**Table 2:** ANOVA results for the quadratic response surface model and optimal conditions for inhibiting *M. plumbeus/racemosus* via the selected yeasts.

	KL371 ^1^	KL874	KL890	KL904	KL1098	KL1351	KL1507	KM364	KM1070	PJ173	PJ433	PJ659	PJ1008	PJ1468
**A: Temperature**	0.001 **	0.409	0.805	0.386	0.536	0.285	0.009 *	0.045 *	0.805	0.015 *	0.000 **	0.005 *	0.011 *	0.014 *
**B: a_w_**	0.025 *	0.409	0.471	0.555	0.536	0.706	0.447	0.650	0.035 *	0.015 *	0.148	0.140	0.213	0.232
**C: pH**	0.175	0.574	0.346	0.175	1.000	1.000	0.1596	0.819	0.346	1.000	0.256	0.245	0.334	0.748
**AA**	0.001 **	0.326	0.619	0.084	0.018 *	0.020 *	0.005 *	0.644	0.070	0.153	0.001 **	0.002 **	0.021 *	0.331
**AB**	0.076	1.000	0.728	0.237	0.391	0.151	1.000	0.526	0.005 *	0.817	0.572	1.000	0.100	0.651
**AC**	1.000	0.688	0.201	0.134	1.000	1.000	0.296	0.747	1.000	0.202	0.281	0.562	0.636	1.000
**BB**	0.008 *	0.609	0.418	0.429	0.886	0.365	0.009 *	0.303	0.014 *	0.853	0.086	0.412	0.939	0.084
**BC**	0.076	0.688	1.000	0.673	1.000	1.000	0.296	0.231	1.000	0.202	0.029 *	1.000	1.000	0.651
**CC**	0.084	0.384	0.619	0.685	0.486	0.670	0.488	0.876	0.418	0.970	0.042 *	0.778	0.228	0.477
***ANOVA (R*** **^2^** ***statistics)***														
**R^2^**	96.88	49.64	52.16	74.93	73.93	77.91	93.13	69.58	91.18	87.07	97.84	92.61	87.58	82.18
**R^2^ adjusted**	91.25	-	-	-	-	-	80.75	-	75.32	63.79	93.95	79.32	65.24	50.11
***Optimal inhibition values***														
**A: Temperature**	10.5	-	-	-	-	-	11.6	-	8.0	15.5	17.3	16.0	14.5	19.8
**B:** **aw**	0.84	-	-	-	-	-	0.97	-	0.84	0.97	0.97	0.97	0.97	0.97
**C: pH**	4.50	-	-	-	-	-	5.50	-	4.80	4.50	5.50	5.50	4.81	5.50
**Estimated value**	3.38	-	-	-	-	-	3.30	-	4.42	5.87	5.56	5.65	5.28	4.67

^1^ The initials belong to the species of the strains: KL (*Kluyveromyces lactis*), KM (*Kluyveromyces marxianus*), and PJ (*Pichia jadinii*). * *p*-values of less than 0.050 are statistically significant (bold type). ** *p*-values of less than 0.001 are statistically significant (bold type).

**Table 3:** Characterisation of the biocontrol mechanisms of selected yeasts: enzymatic activities (pectinase, β-glucanase, chitinase, and protease), antibiosis, biofilm formation, and parasitism.

Strains	Enzymatic Activities					Biofilm Formation	Antibiosis	Parasitism		
	Pectinase	β-Glucanase	Quitinase	Protease 25 °C	Protease 15 °C			PC	FV	MP/R
**GC663 ^1^**	+++ ^2^	+++	−	−	−	−	−	+^3^	+	+
**KL371**	−	−	−	−	+	−	−	−	−	−
**KL874**	−	−	−	−	++	−	−	−	−	−
**KL1098**	−	−	−	++	+	−	−	−	−	−
**KL890**	−	−	−	−	++	−	−	−	−	−
**KL904**	−	−	−	−	+	−	−	−	−	+
**KL1351**	−	−	−	++	++	−	−	−	−	−
**KL1507**	−	−	−	++	+++	−	−	+	−	−
**KM364**	+	−	−	−	−	−	−	−	−	−
**KM1070**	−	−	−	−	−	++	−	−	−	−
**PJ173**	−	+	−	−	−	−	−	−	−	+
**PJ433**	−	++	−	++	++	−	−	+	+	−
**PJ659**	−	++	−	−	−	−	−	−	−	−
**PJ1008**	++	++	−	−	−	−	−	−	+	−
**PJ1468**	−	+	−	+	+	−	−	−	+	−

^1^ The initials belong to the species of the strains: GC (*Geotrichum candidum*), KL (*Kluyveromyces lactis*), KM (*Kluyveromyces marxianus*), PJ (*Pichia jadinii*), PC (*Penicillium commune*), FV (*Fusarium verticillioides*), and MP/R (*Mucor plumbeus/racemosus*). ^2^ The symbol ‘+’ indicates the intensity of the activity for enzymatic activities and biofilm formation: + (low intensity), ++ (moderate intensity), and +++ (high intensity). ^3^ The symbol ‘+’ or ‘−’ indicates the ability of the yeast strain for antibiosis and parasitism of the hyphae of the target moulds: − (negative activity) and + (positive activity).

## Data Availability

The original contributions presented in the study are included in the article/supplementary material; further inquiries can be directed to the corresponding author.

## Supplementary Materials

The following supporting information can be downloaded at: https://www.mdpi.com/article/10.3390/foods14142446/s1. Table S1. yeast and mould isolates from PDO “Torta del Casar” and “Queso de la Serena” cheeses selected for this study. Table S2. conditions of temperature, a_w_ and pH for modelling antagonist yeast activity under the cheese ripening process. Table S3. Descriptive statistics for the size of inhibition halo (radio: mm) of selected antagonistic yeasts across the range of conditions tested in the experimental model. Figure S1. Microscope image of the adhesion of *Geotrichum candidum* GC663 to the three target moulds evaluated: *Penicillium commune* (A), *Fusarium verticillioides* (B), and *Mucor plumbeus/racemosus* (C). Figure S2. Growth inhibition percentage of *Penicillium commune* when exposed to volatile organic compounds (VOCs) produced by the selected yeasts. Figure S3. Spore germination inhibition percentage of *Penicillium commune* when exposed to confrontation to the selected yeasts. Figure S4. Growth inhibition percentage of *Penicillium commune* when exposed to eight yeast strains during the 11 days of confrontation on soft cheese wedges. Figure S5. Image of confrontations of *Geotrichum candidum* GC663 against *Fusarium verticillioides* (A), and *Mucor plumbeus/racemosus* (B) on cheese wedges at 9 days of incubation. Figure S6. Image of confrontations of *Pichia jadinii* PJ433 against *Penicillium commune* (A), *Fusarium verticillioides* (B), and *Mucor plumbeus/racemosus* (C) on cheese wedges at 9 days of incubation.

## References

[1] Walsh A.M., Macori G., Kilcawley K.N., Cotter P.D. (2020). Meta-analysis of cheese microbiomes highlights contributions to multiple aspects of quality. Nat. Food.

[2] Kure C.F., Skaar I. (2019). The fungal problem in cheese industry. Curr. Opin. Food Sci..

[3] Garnier L., Valence F., Mounier J. (2017). Diversity and control of spoilage fungi in dairy products: An update. Microorganisms.

[4] Ledenbach L.H., Marshall R.T. (2009). Microbiological spoilage of dairy products. Compendium of the Microbiological Spoilage of Foods and Beverages.

[5] Hymery N., Vasseur V., Coton M., Mounier J., Jany J.L., Barbier G., Coton E. (2014). Filamentous fungi and mycotoxins in cheese: A review. Compr. Rev. Food Sci. Food Saf..

[6] Shi C., Maktabdar M. (2022). Lactic acid bacteria as biopreservation against spoilage moulds in dairy products—A review. Front. Microbiol..

[7] Finol M.L., Marth E.H., Lindsay R.C. (1982). Depletion of sorbate from different media during growth of *Penicillium* species. J. Food Prot..

[8] Sofos J.N., Busta F.F., Davidson P.M., Branen A.L. (1993). Sorbic acid and sorbates. Antimicrobials in Foods.

[9] Marth E.H., Capp C.M., Hasenzahl L., Jackson H.W., Hussong R.V. (1966). Degradation of potassium sorbate by *Penicillium* species. J. Dairy Sci..

[10] Suhr K.I., Nielsen P.V. (2004). Effect of weak acid preservatives on growth of bakery product spoilage fungi at different water activities and pH values. Int. J. Food Microbiol..

[11] Davidson P.M., Doan C. (2020). Natamycin. Antimicrobials in Food.

[12] O’Sullivan K. (2024). Recombinant Expression and Purification of Natamycinase: A Novel Serine Esterase Capable of Degrading the Polyene Antibiotic Natamycin. Master’s Thesis.

[13] Maruyama S., Streletskaya N.A., Lim J. (2021). Clean label: Why this ingredient but not that one?. Food Qual. Prefer..

[14] Salas M.L., Mounier J., Maillard M.B., Valence F., Coton E., Thierry A. (2019). Identification and quantification of natural compounds produced by antifungal bioprotective cultures in dairy products. Food Chem..

[15] Garnier L., Penland M., Thierry A., Maillard M.B., Jardin J., Coton M., Mounier J. (2020). Antifungal activity of fermented dairy ingredients: Identification of antifungal compounds. Int. J. Food Microbiol..

[16] Liang N., Zhao Z., Curtis J.M., Gänzle M.G. (2022). Antifungal cultures and metabolites of lactic acid bacteria for use in dairy fermentations. Int. J. Food Microbiol..

[17] Souza L.V., Martins E., Moreira I.M.F.B., De Carvalho A.F. (2022). Strategies for the development of bioprotective cultures in food preservation. Int. J. Microbiol..

[18] Freimoser F.M., Rueda-Mejia M.P., Tilocca B., Migheli Q. (2019). Biocontrol yeasts: Mechanisms and applications. World J. Microbiol. Biotechnol..

[19] Fröhlich-Wyder M.T., Arias-Roth E., Jakob E. (2019). Cheese yeasts. Yeast.

[20] Hernández A., Rodríguez A., Córdoba M.G., Martín A., Ruiz-Moyano S. (2022). Fungal control in foods through biopreservation. Curr. Opin. Food Sci..

[21] Huang C., Zhang L., Johansen P.G., Petersen M.A., Arneborg N., Jespersen L. (2021). *Debaryomyces hansenii* strains isolated from Danish cheese brines act as biocontrol agents to inhibit germination and growth of contaminating moulds. Front. Microbiol..

[22] Alvarez M., Delgado J., Nunez F., Roncero E., Andrade M.J. (2022). Proteomic approach to unveil the ochratoxin A repression by *Debaryomyces hansenii* and rosemary on *Penicillium nordicum* during dry-cured fermented sausages ripening. Food Control.

[23] Liu S.Q., Tsao M. (2009). Biocontrol of dairy moulds by antagonistic dairy yeast Debaryomyces hansenii in yoghurt and cheese at elevated temperatures. Food Control.

[24] Merchan A.V., Benito M.J., Galván A.I., Ruiz-Moyano S. (2020). Identification and selection of yeast with functional properties for future application in soft paste cheese. LWT.

[25] Moure M.C., Pérez-Torrado R., Garmendia G., Vero S., Querol A., Alconada T., León Peláez Á. (2023). Characterization of kefir yeasts with antifungal capacity against Aspergillus species. Int. Microbiol..

[26] Zheng X., Zheng L., Xia F., Li J., Zhou W., Yuan L., Yang Z. (2023). Biological control of blue mould rot in apple by *Kluyveromyces marxianus* XZ1 and the possible mechanisms of action. Postharvest Biol. Technol..

[27] Van den Tempel T., Jakobsen M. (2000). The technological characteristics of *Debaryomyces hansenii* and *Yarrowia lipolytica* and their potential as starter cultures for production of Danablu. Int. Dairy J..

[28] Lessard M.H., Bélanger G., St-Gelais D., Labrie S. (2012). The composition of Camembert cheese-ripening cultures modulates both mycelial growth and appearance. Appl. Environ. Microbiol..

[29] Merchan A.V., Ruiz-Moyano S., Hernández M.V., Benito M.J., Aranda E., Rodríguez A., Martín A. (2022). Characterization of autochthonal yeasts isolated from Spanish soft raw ewe milk protected designation of origin cheeses for technological application. J. Dairy Sci..

[30] White T.J., Bruns T., Lee S.J., Taylor J., Innis M.A., Gelfand D.H., Sninsky J.J., White T.J. (1990). Amplification and direct sequencing of fungal ribosomal RNA genes for phylogenetics. PCR Protocols: A Guide to Methods and Applications.

[31] Glass N.L., Donaldson G.C. (1995). Development of primer sets designed for use with the PCR to amplify conserved genes from filamentous ascomycetes. Appl. Environ. Microbiol..

[32] O’Donnell K., Reynolds D.R., Taylor J.W. (1993). The fungal holomorph: Mitotic, meiotic and pleomorphic speciation in fungal systematics. The Fungal Holomorph.

[33] Cabañas C.M., Hernández A., Martínez A., Tejero P., Vázquez-Hernández M., Martín A., Ruiz-Moyano S. (2020). Control of *Penicillium glabrum* by indigenous antagonistic yeast from vineyards. Foods.

[34] Casquete R., Benito M.J., Córdoba M.G., Ruiz-Moyano S., Galván A.I., Martín A. (2018). Physicochemical factors affecting the growth and mycotoxin production of *Penicillium* strains in a synthetic cheese medium. LWT.

[35] Freitas A.C., Macedo A.C., Malcata F.X. (2000). Technological and organoleptic issues pertaining to cheeses with denomination of origin manufactured in the Iberian Peninsula from ovine and caprine milks. Food Sci. Technol. Int..

[36] Trmčić A., Ralyea R., Meunier-Goddik L., Donnelly C., Glass K., D’amico D., Wiedmann M. (2017). Consensus categorization of cheese based on water activity and pH—A rational approach to systemizing cheese diversity. J. Dairy Sci..

[37] Cordero-Bueso G., Mangieri N., Maghradze D., Foschino R., Valdetara F., Cantoral J.M., Vigentini I. (2017). Wild grape-associated yeasts as promising biocontrol agents against *Vitis vinifera* fungal pathogens. Front. Microbiol..

[38] Ruiz-Moyano S., Hernández A., Galván A.I., Córdoba M.G., Casquete R., Serradilla M.J., Martín A. (2020). Selection and application of antifungal VOCs-producing yeasts as biocontrol agents of grey mould in fruits. Food Microbiol..

[39] Kolde R. pheatmap: Pretty Heatmaps. R Package Version 1.0.12. https://CRAN.R-project.org/package=pheatmap.

[40] Chen X., Abdallah M.F., Landschoot S., Audenaert K., De Saeger S., Rajkovic A. (2023). *Aspergillus flavus* and *Fusarium verticillioides* and their main mycotoxins: Global distribution and scenarios of interactions in maize. Toxins.

[41] Marín P., Palmero D., Jurado M. (2015). Occurrence of moulds associated with ovine raw milk and cheeses of the Spanish region of Castilla La Mancha. Int. J. Dairy Technol..

[42] Arispe-Vazquez J.L., Sanchez-Arizpe A., Galindo-Cepeda M.E. (2021). Microbiota fúngica en quesos artesanales en Saltillo, Coahuila, México. Rev. Ion.

[43] Sipiczki M. (2023). Identification of antagonistic yeasts as potential biocontrol agents: Diverse criteria and strategies. Int. J. Food Microbiol..

[44] Fiori S., Urgeghe P.P., Hammami W., Razzu S., Jaoua S., Migheli Q. (2014). Biocontrol activity of four non-and low-fermenting yeast strains against *Aspergillus carbonarius* and their ability to remove ochratoxin A from grape juice. Int. J. Food Microbiol..

[45] Ghanbari R., Rezaie S., Noorbakhsh F., Khaniki G.J., Soleimani M., Aghaee E.M. (2019). Biocontrol effect of *Kluyveromyces lactis* on aflatoxin expression and production in *Aspergillus parasiticus*. FEMS Microbiol. Lett..

[46] Alasmar R., Ul-Hassan Z., Zeidan R., Al-Thani R., Al-Shamary N., Alnaimi H., Jaoua S. (2020). Isolation of a novel *Kluyveromyces marxianus* strain QKM-4 and evidence of its volatilome production and binding potentialities in the biocontrol of toxigenic fungi and their mycotoxins. ACS Omega.

[47] Ramos-Pereira J., Mareze J., Fernández D., Rios E.A., Santos J.A., López-Díaz T.M. (2021). Antifungal activity of lactic acid bacteria isolated from milk against *Penicillium commune*, *P. nordicum*, and *P. verrucosum*. Int. J. Food Microbiol..

[48] Mareze J., Ramos-Pereira J., Santos J.A., Beloti V., López-Díaz T.M. (2022). Identification and characterisation of lactobacilli isolated from an artisanal cheese with antifungal and antibacterial activity against cheese spoilage and mycotoxigenic *Penicillium* spp. *Int*. Dairy J..

[49] Salas M.L., Thierry A., Lemaitre M., Garric G., Harel-Oger M., Chatel M., Coton E. (2018). Antifungal activity of lactic acid bacteria combinations in dairy mimicking models and their potential as bioprotective cultures in pilot scale applications. Front. Microbiol..

[50] Garnier L., Mounier J., Lê S., Pawtowski A., Pinon N., Camier B., Valence F. (2019). Development of antifungal ingredients for dairy products: From in vitro screening to pilot scale application. Food Microbiol..

[51] Medina-Córdova N., Rosales-Mendoza S., Hernández-Montiel L.G., Angulo C. (2018). The potential use of *Debaryomyces hansenii* for the biological control of pathogenic fungi in food. Biol. Control.

[52] Théolier J., Hammami R., Labelle P., Fliss I., Jean J. (2013). Isolation and identification of antimicrobial peptides derived by peptic cleavage of whey protein isolate. J. Funct. Foods.

[53] Hidalgo M.E., Côrrea A.P.F., Canales M.M., Daroit D.J., Brandelli A., Risso P. (2015). Biological and physicochemical properties of bovine sodium caseinate hydrolysates obtained by a bacterial protease preparation. Food Hydrocoll..

[54] Sui Y., Wisniewski M., Droby S., Liu J. (2015). Responses of yeast biocontrol agents to environmental stress. Appl. Environ. Microbiol..

[55] Hudecová A., Valik L., Liptakova D. (2009). Influence of temperature on the surface growth of *Geotrichum candidum*. Acta Chim. Slovaca.

[56] Spadaro D., Droby S. (2016). Development of biocontrol products for postharvest diseases of fruit: The importance of elucidating the mechanisms of action of yeast antagonists. Trends Food Sci. Technol..

[57] Ruiz-Moyano S., Martín A., Villalobos M.C., Calle A., Serradilla M.J., Córdoba M.G., Hernández A. (2016). Yeasts isolated from figs (*Ficus carica* L.) as biocontrol agents of postharvest fruit diseases. Food Microbiol..

[58] Jensen A.N., Jensen L.T., Costa L.G., Aschner M. (2014). Manganese transport, trafficking and function in invertebrates. Manganese in Health and Disease.

[59] Krause D.J., Kominek J., Opulente D.A., Shen X.X., Zhou X., Langdon Q.K., Hittinger C.T. (2018). Functional and evolutionary characterization of a secondary metabolite gene cluster in budding yeasts. Proc. Natl. Acad. Sci. USA.

[60] Siedler S., Rau M.H., Bidstrup S., Vento J.M., Aunsbjerg S.D., Bosma E.F., Neves A.R. (2020). Competitive exclusion is a major bioprotective mechanism of lactobacilli against fungal spoilage in fermented milk products. Appl. Environ. Microbiol..

[61] Ning M., Guo Q., Guo P., Cui Y., Wang K., Du G., Yue T. (2025). Biocontrol activity of *Kluyveromyces marxianus* YG-4 against *Penicillium expansum* LPH9 on apples. Int. J. Food Microbiol..

[62] Choińska R., Piasecka-Jóźwiak K., Chabłowska B., Dumka J., Łukaszewicz A. (2020). Biocontrol ability and volatile organic compounds production as a putative mode of action of yeast strains isolated from organic grapes and rye grains. Antonie Leeuwenhoek.

[63] Tejero P., Martín A., Rodríguez A., Galván A.I., Ruiz-Moyano S., Hernández A. (2021). In vitro biological control of *Aspergillus flavus* by *Hanseniaspora opuntiae* L479 and *Hanseniaspora uvarum* L793, producers of antifungal volatile organic compounds. Toxins.

[64] Farbo M.G., Urgeghe P.P., Fiori S., Marcello A., Oggiano S., Balmas V., Migheli Q. (2018). Effect of yeast volatile organic compounds on ochratoxin A-producing *Aspergillus carbonarius* and *A. ochraceus*. Int. J. Food Microbiol..

[65] Hassan Z.U., Al Thani R., Atia F.A., Alsafran M., Migheli Q., Jaoua S. (2021). Application of yeasts and yeast derivatives for the biological control of toxigenic fungi and their toxic metabolites. Environ. Technol. Innov..

[66] Borges F., Briandet R., Callon C., Champomier-Vergès M.C., Christieans S., Chuzeville S., Zagorec M. (2022). Contribution of omics to biopreservation: Toward food microbiome engineering. Front. Microbiol..

[67] Coni E., Bocca A., Ianni D., Caroli S. (1995). Preliminary evaluation of the factors influencing the trace element content of milk and dairy products. Food Chem..

