# Supplementary material for: Biocontrol of Cheese Spoilage Moulds Using Native Yeasts
Source: Foods. 2025 Jul 11;14(14):2446. doi: 10.3390/foods14142446 (PMC12294727; doi:10.3390/foods14142446)

Biocontrol of cheese spoilage moulds by autochthonous yeast

Catalina M. Cabañas, Alejandro Hernández León, Santiago Ruiz-Moyano*, Almudena V. Merchán, José Manuel Martínez Torres, Alberto Martín.

SUPPLEMENTAL MATERIAL

This article contains three supplementary Tables and six Figures

Table S1: yeast and mould isolates from PDO "Torta del Casar" and "Queso de la Serena" cheeses selected for this study (Merchan et al., 2022).

Type microorganism	Genus	Especies	Nº of isolates selected	Code strain
Yeast	*Candida*	*C. zeylanoides*	9	17, 350, 665, 882, 896, 921, 925, 1214, 1459
	Debaryomyces	*D. hansenii*	16	329, 330, 353, 898, 910, 923, 933, 954, 1088, 1092, 1100, 1206, 1232, 1238, 1249, 1251
	Geotrichum	*G. candidum*	5	369, 663, 902, 1006, 1461
	Kluyveromyces	*K. lactis*	7	371, 874, 890, 904, 1098, 1351, 1507
		K. marxianus	2	364, 1070
	Pichia	*P. fermentans*	4	170, 1076, 1364, 1438
		P. jadinii	5	173, 433, 659, 1008, 1468
		P. kudriavzevii	16	2, 165, 373, 435, 645, 871, 939, 1074, 1094, 1193, 1224, 1241, 1360, 1368, 1436, 1509
		P. sporocuriosa	1	1443
	Yarrowia	*Y. alimentaria*	4	929, 1204, 1221, 1476,
		Y. lipolytica	15	387, 498, 502, 510, 522, 667, 888, 912, 918, 947, 996, 1090, 1160, 1202, 1362
	Total: 6	*Total:* 11	*Total:* 84	-
Mould	*Mucor*	*M. plumbeus/racemosus*	1	2367
	Penicillium	*P. commune*	1	1031
	Fusarium	*F. verticillioides*	1	1191
	Total: 3	*Total: 3*	*Total: 3*	-

Table S2: conditions of temperature, a_w and pH for modelling antagonist yeast activity under the cheese ripening process.

**Block**	**Temperature**	**pH**	**a_w**
1	20	4.5	0.905
2	20	5	0.97
3	20	5	0.84
4	20	5.5	0.905
5	14	4.5	0.97
6	14	4.5	0.84
7	14	5	0.905
8	14	5.5	0.97
9	14	5.5	0.84
10	8	4.5	0.905
11	8	5	0.97
12	8	5	0.84
13	8	5.5	0.905
14	14	5	0.905
15	14	5	0.905

Table S3: Descriptive statistics for the size of inhibition halo (radio: mm) of selected antagonistic yeasts across the range of conditions tested in the experimental model.

	F. verticillioides				*M. plumbeus/racemosus*			
	Mean	SD	Min	Max	Mean	SD	Min	Max
**KL371**[1]	1.80 ± 0.68		0	3	2.00 ± 0.76		1	3
**KL874**	1.73 ± 0.70		1	3	1.73 ± 0.50		1	2.5
**KL890**	1.80 ± 0.68		0	3	2.17 ± 0.59		1	3
**KL904**	1.60 ± 1.18		0	3	1.87 ± 0.67		0	3
**KL1098**	1.80 ± 0.56		1	3	2.57 ± 0.62		2	4
**KL1351**	1.33 ± 0.90		0	3	2.43 ± 0.56		1.5	3
**KL1507**	1.67 ± 0.90		0	3	1.67 ± 0.98		0	3
**KM364**	0.67 ± 0.62		0	2	0.83 ± 0.79		0	2
**KM1070**	0.93 ± 0.96		0	3	0.97 ± 1.37		0	5
**PJ173**	2.53 ± 1.51		0	4	3.23 ± 1.70		0	5.5
**PJ433**	2.87 ± 1.06		1	4	3.00 ± 1.68		0	5
**PJ659**	2.20 ± 1.21		0	4	3.00 ± 1.77		0	5
**PJ1008**	2.20 ± 1.15		0	4	3.40 ± 1.68		0	5
**PJ1468**	1.60 ± 1.06		0	4	2.20 ± 1.47		0	4

[1]The initials belong to the species of the strains: KL (*Kluyveromyces lactis*), KM (*Kluyveromyces marxianus*) and PJ (*Pichia jadinii*).

Figure S1: Microscope image of the adhesion of *Geotrichum candidum* GC663 to the three target moulds evaluated: *Penicillium commune* (A), *Fusarium verticillioides* (B), and *Mucor plumbeus/racemosus* (C).

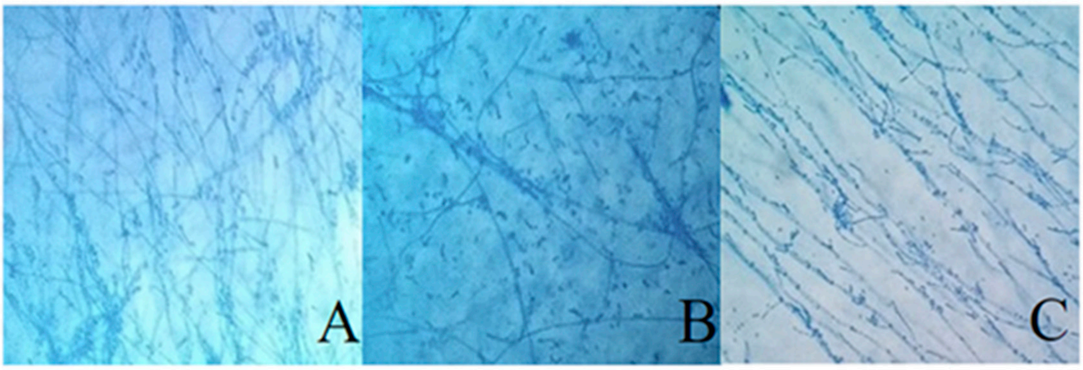

Figure S2: Growth Inhibition percentage of *Penicillium commune* when exposed to volatile organic compounds (VOCs) produced by the selected yeasts. On the ordinate axis, positive values mean reduced growth, and negative values mean increased mycelial growth. Species are distinguished by colour: green, *Geotrichum candidum*; lavender, *Kluyveromyces lactis*; yellow, *Kluyveromyces marxianus*; orange, *Pichia jadinii*. * Indicates yeasts with significant activity ($p \leq 0.05$). Error bars correspond to the 95% confidence interval from Tukey's honestly significant difference test (±12.68).

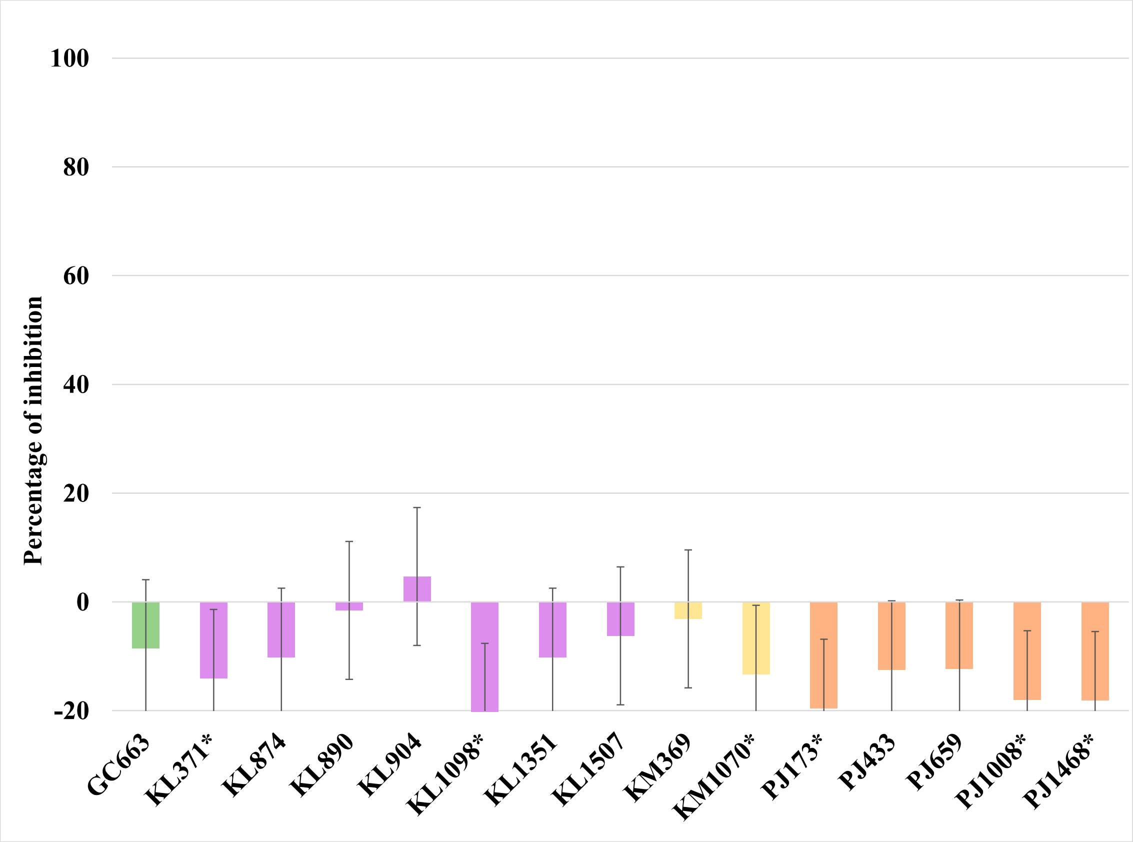

Figure S3: Spore germination inhibition percentage of *Penicillium commune* when exposed to confrontation to the selected yeasts. On the ordinate axis, positive values mean reduced germination, negative values mean increased germination. Species are distinguished by colour: green, *Geotrichum candidum*; lavender, *Kluyveromyces lactis*; yellow, *Kluyveromyces marxianus*; orange, *Pichia jadinii*. * Indicates yeasts with significant activity ($p \leq 0.05$). Error bars correspond to the 95% confidence interval from Tukey's honestly significant difference test (±6.9).

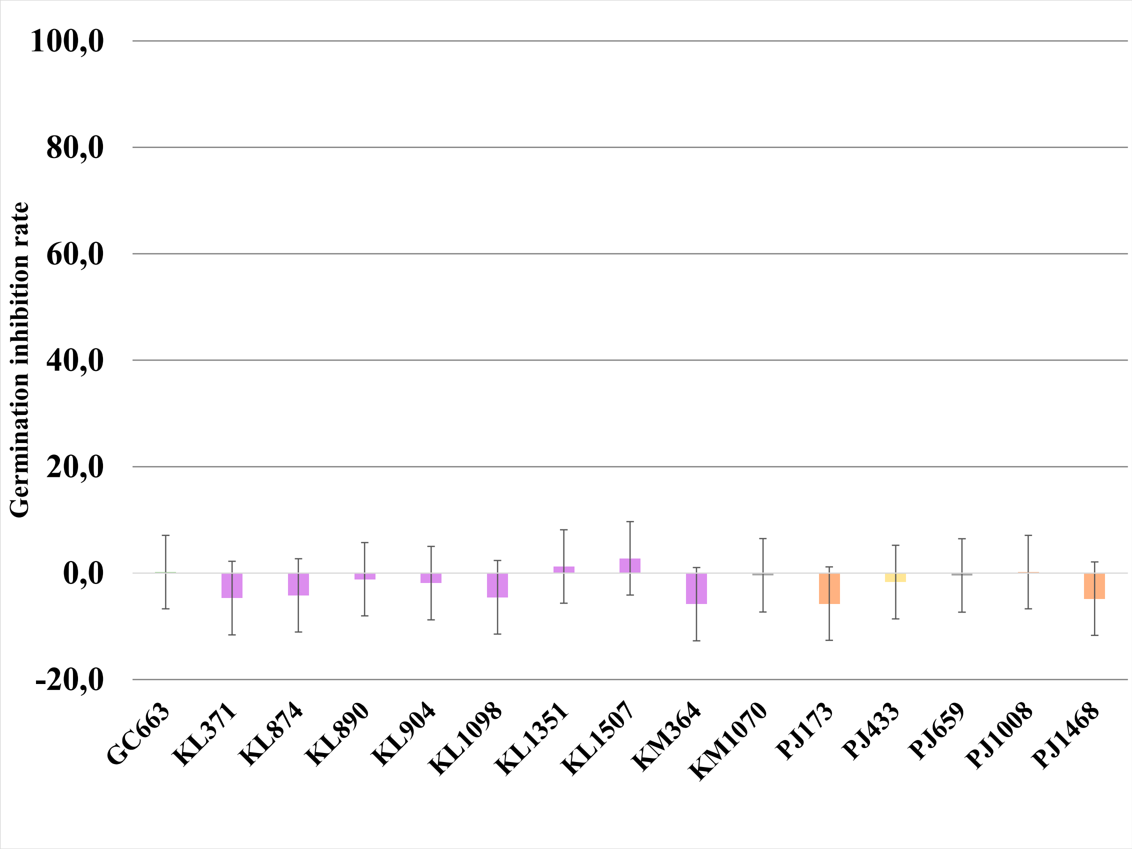

Figure S4: Growth Inhibition percentage of *Penicillium commune* when exposed to eight yeast strains during the 11 days of confrontation on soft cheese wedges. Species are distinguished by colour: in blue, the positive controls Nam = natamycin (1000 mg/L); in green, *Geotrichum candidum*; in lavender, *Kluyveromyces lactis*; in yellow, *Kluyveromyces marxianus*; orange, *Pichia jadinii*. The different growth days are shown with various textures. * Indicates the yeast strains that produced significant inhibition ($p \leq 0.05$) in at least 2 storage days. The 95% confidence interval from Tukey's honestly significant difference test was ±7.31.

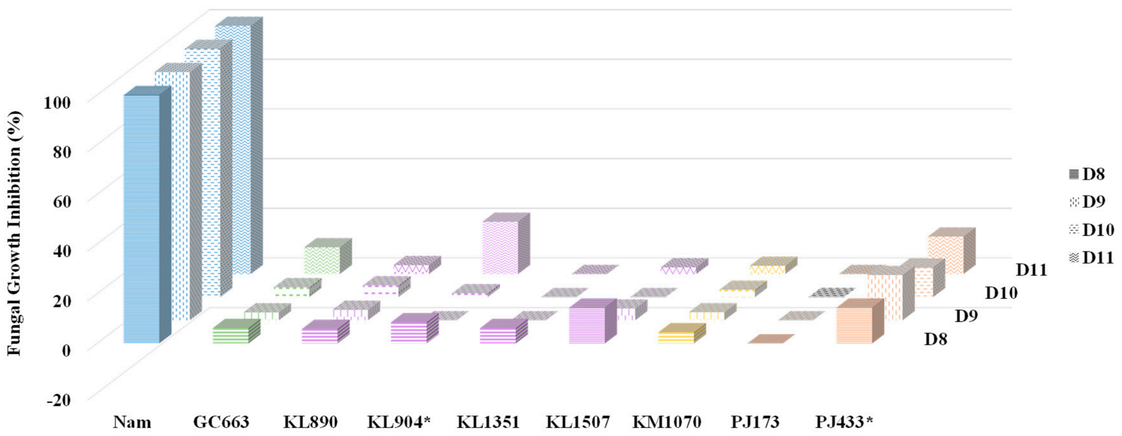

Figure S5: Image of confrontations of *Geotrichum candidum* GC663 against *Fusarium verticillioides* (A), and *Mucor plumbeus/racemosus* (B) on cheese wedges at 9 days of incubation. Control and 'Nam' represent target mould growth without yeast confrontation and with natamycin treatment with solution at 1000 mg/L, respectively.

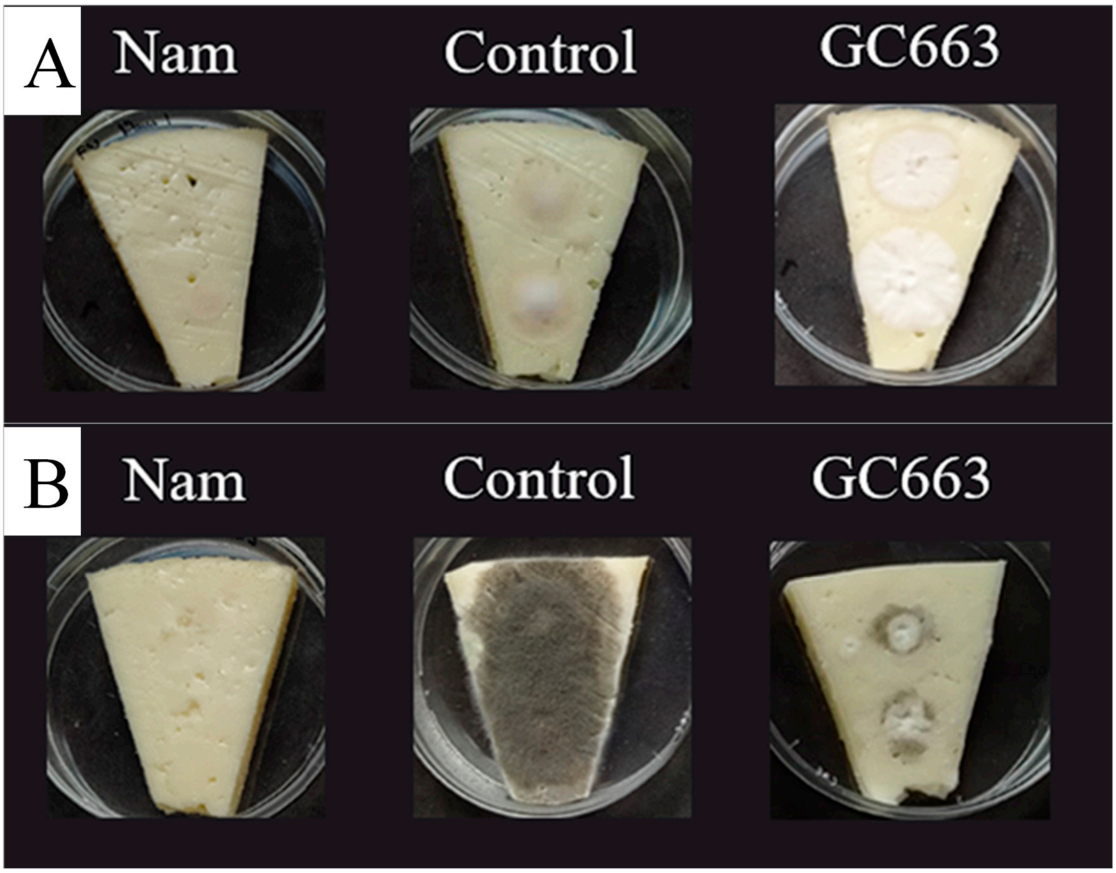

Figure S6: Image of confrontations of *Pichia jadinii* PJ433 against *Penicillium commune* (A), *Fusarium verticillioides* (B), and *Mucor plumbeus/racemosus* (C) on cheese wedges at 9 days of incubation. Control and 'Nam' represent target mould growth without yeast confrontation and with natamycin treatment with solution at 1000 mg/L, respectively.

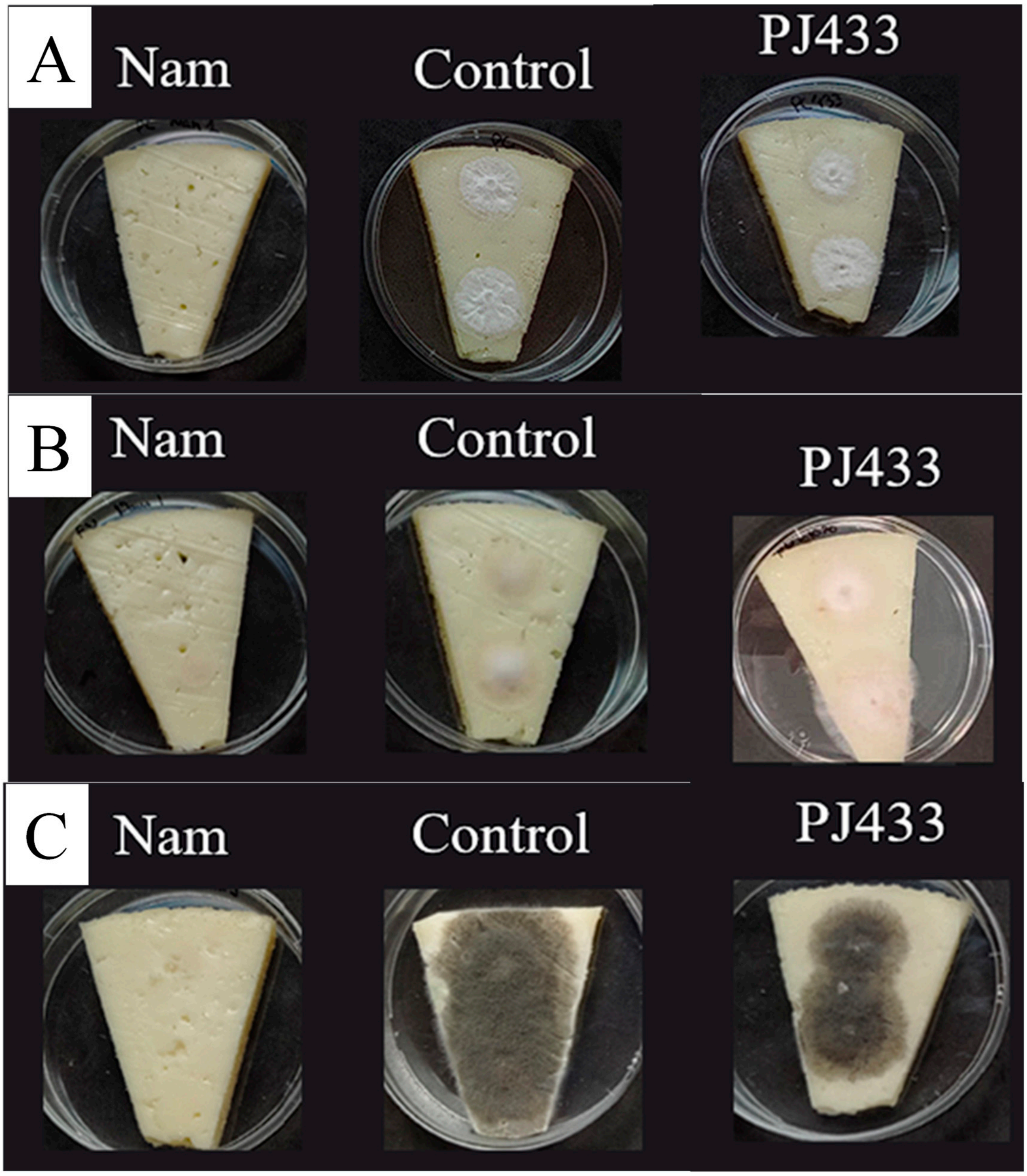

Supplement: Supplementary file 1 [file foods-14-02446-s001.zip › foods-3700265-supplementary.pdf]
